# Fundamentals of microbiome-based therapies for reproductive tract inflammatory diseases in domestic animals

**DOI:** 10.1590/1984-3143-AR2025-0030

**Published:** 2025-09-01

**Authors:** Uxía Yáñez Ramil, Sylwia Jezierska, Milena Krupa, Osvaldo Bogado Pascottini

**Affiliations:** 1 School of Veterinary Medicine, University College Dublin, Dublin, Ireland; 2 Unidad de Reproducción y Obstetricia Veterinaria, Departamento de Patología Animal, Facultad de Veterinaria, Universidade de Santiago de Compostela, Lugo, Spain; 3 Insight Research Ireland Centre for Data Analytics, Dublin City University, Dublin, Ireland

**Keywords:** uterine disease, therapeutics, antibiotic resistance, microbiota, microbiome transfer, synthetic microbiome

## Abstract

Reproductive tract inflammatory diseases (RTID) present significant health challenges in domestic animals, impacting welfare, fertility, and productivity. Traditionally, antibiotics have been the primary treatment for these conditions, however, the rise of antimicrobial resistance calls for alternative approaches. The microbiome of the female reproductive tract plays a vital role in maintaining reproductive health, and emerging evidence suggests that microbiome-based therapies, such as ‘natural’ or ‘synthetic’ microbiome transplantation, may offer sustainable solutions for RTID management. This review explores the composition and dynamics of the reproductive microbiome in both healthy and diseased states in cows, mares, sows, dogs, and cats. It also examines current treatments and the potential for microbiome-based interventions to replace or complement antibiotic therapies. Although research on microbiome-based therapies for preventing or treating RTID in domestic animals is virtually non-existent, vaginal and uterine microbiomes transplantation in mice and women show promise but require further investigation to evaluate their efficacy and safety across species with varying reproductive physiologies. Additionally, synthetic microbiome therapies present a controlled and reproducible alternative, though they face challenges in design, engraftment, and regulatory approval. The transition from antibiotic dependence to microbiome-based solutions marks a paradigm shift in veterinary medicine, but successful implementation demands a deeper understanding of host-microbiome interactions, rigorous safety protocols, and species-specific research.

## Introduction

Optimal reproductive health plays a crucial role in domestic species, profoundly influencing their welfare, sustainability, profitability, and population management. In livestock such as cattle and pigs, reproductive efficiency is essential for meeting production demands and ensuring economic stability for farmers. Fertility challenges, for instance, can lead to significant financial losses due to reduced calving or farrowing rates, prolonged intervals between births, and increased culling rates. For equine populations, maintaining reproductive health is key to preserving genetic diversity and performance potential. Similarly, in companion animals like dogs and cats, reproductive health directly affects individual well-being and promotes responsible breeding practices.

A critical disruptor of reproductive health is dysbiosis, defined as an imbalance in the composition of beneficial microbial communities compared to those in healthy individuals (Walker, 2017). In the reproductive tract, this microbial ecosystem imbalance leads to infection, inflammation, and conditions that compromise health and fertility. In cows and sows, uterine infections such as metritis and endometritis, frequently linked to dysbiosis, are major contributors to infertility (Pascottini et al., 2023a). In mares, altered uterine microbiota can hinder conception and pregnancy success (Canisso et al., 2020), while in dogs and cats, dysbiosis-related conditions like pyometra often require urgent medical care (Verstegen et al., 2008).

For decades, microbial presence in the female reproductive tract has been associated with inflammation, disease, and poor pregnancy outcomes. Today, we recognize that healthy individuals host diverse microbial communities in different body sites including the skin, oral cavity, respiratory, gastrointestinal, and reproductive tracts (Khan et al., 2024). These communities comprising bacteria, viruses, fungi, and archaea, together with their theatre of activity are collectively called microbiome. Functioning in close symbiosis with the host, the microbiome plays a crucial role in supporting essential biological processes. The female reproductive tract harbours its own dynamic ecosystem, where chemicals, immune components, host cells, and microbes interact to maintain balance (Gholiof et al., 2022). While the microbiome’s role in reproductive health is less understood than its gut counterpart, emerging evidence highlights its importance in safeguarding against infections, enhancing immune resilience, supporting embryo implantation, and pregnancy success (Golińska et al., 2021; Zhang et al., 2021; France et al., 2022; Khan et al., 2024).

In domestic animals, reproductive tract inflammatory diseases (RTID) are predominantly treated with antimicrobials. However, the urgent need for alternatives is clear: projections suggest antibiotic resistance could claim nearly 40 million lives globally by 2050 if no action is taken to stop this trend (Naddaf, 2024). This crisis, fuelled by indiscriminate antimicrobial use, threatens both public health and food security. The World Health Organization (WHO) now classifies 15 families of antibiotic-resistant bacteria as critical human health risks (WHO, 2024) and advises against the use of antimicrobials for growth promotion or disease prevention in agriculture. While RTID in animals is rarely fatal, its impact on reproductive performance drives reliance on antimicrobial therapies. Innovative yet not conclusive research is shifting toward sustainable, non-antibiotic solutions to manage these conditions.

Advances in understanding host-microbiome interactions have paved the way for pioneering therapies aimed at restoring microbial balance and improving health. Among these, microbiome transplantation—transferring microbial communities from healthy donors to diseased recipients—shows promise by leveraging the microbiome’s natural role in disease prevention and health promotion. The objective of this narrative review is to provide insight into the composition of the female reproductive tract microbiome in domestic animals in health and disease and discuss current treatments for RTID. Building on this foundation, we aim to explore the future of RTID management through microbiome-based approaches, including microbiome transplantation and synthetic microbiome therapies, while addressing the challenges and opportunities in translating these advances into practical veterinary applications.

## Diversity matters: variations within the healthy reproductive tract microbiome

**Figure 1:**
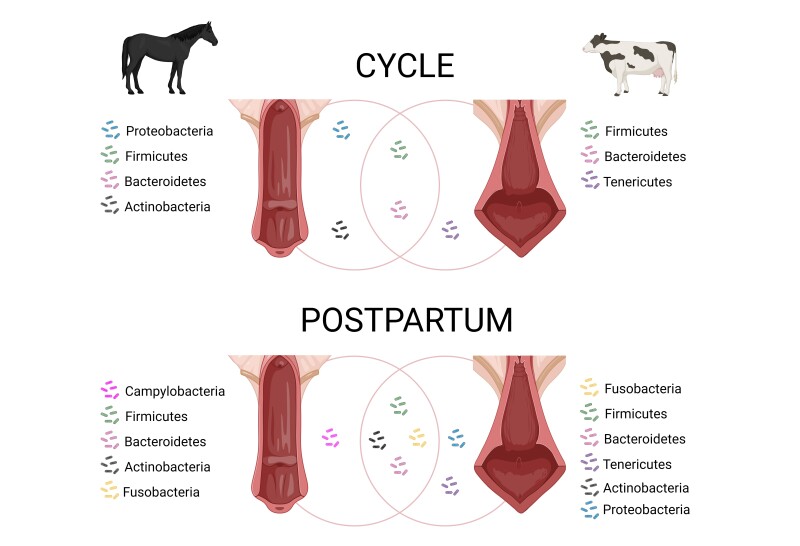
Graphical representation of the most common bacterial phyla identified in the equine and bovine vagina via 16S rRNA sequencing at different physiological stages. Cycle: healthy, cycling adult mares (5-23 yo) at oestrus and dioestrus, and healthy, cycling virgin heifers (13-16 mo) at oestrus and dioestrus. Postpartum: mares ~12 h after foaling and cows ≤ 7 d after calving. The bacteria located in the intersection between both circles represent the common phyla between species. Adapted from Bicalho et al. (2017a), Barba et al. (2020), Quereda et al. (2020) and Płoneczka-Janeczko et al. (2024).

**Figure 2:**
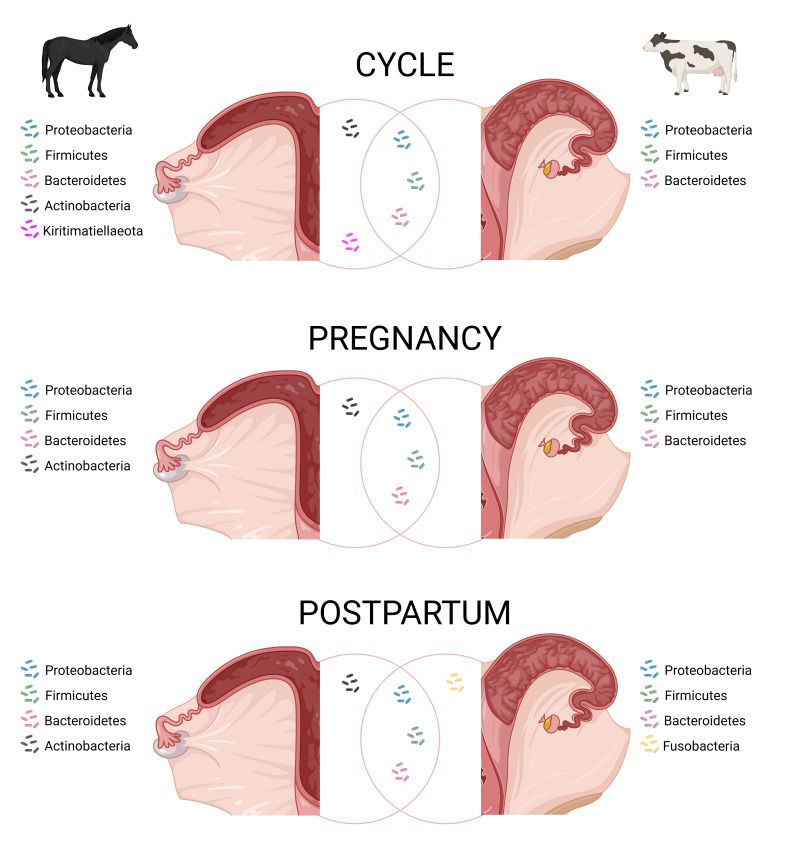
**Figure sho**wing the most common bacterial phyla identified in the equine and bovine uterus via 16S rRNA sequencing at different physiological stages. Cycle: healthy, cycling (information about the moment of oestrus cycle was not available) adult mares (4-18 yo), and healthy, cycling virgin heifers (14 mo) at oestrus. Pregnancy: mares ~280 d of pregnancy and cows at last trimester of pregnancy. Postpartum: mares just after foaling and cows 3-12 d after calving. The bacteria located in the intersection between both circles represent the common phyla between species. Adapted from Moore et al. (2017), Bicalho et al. (2017b), Holyoak et al. (2022) and van Heule et al. (2023).

**Figure 3:**
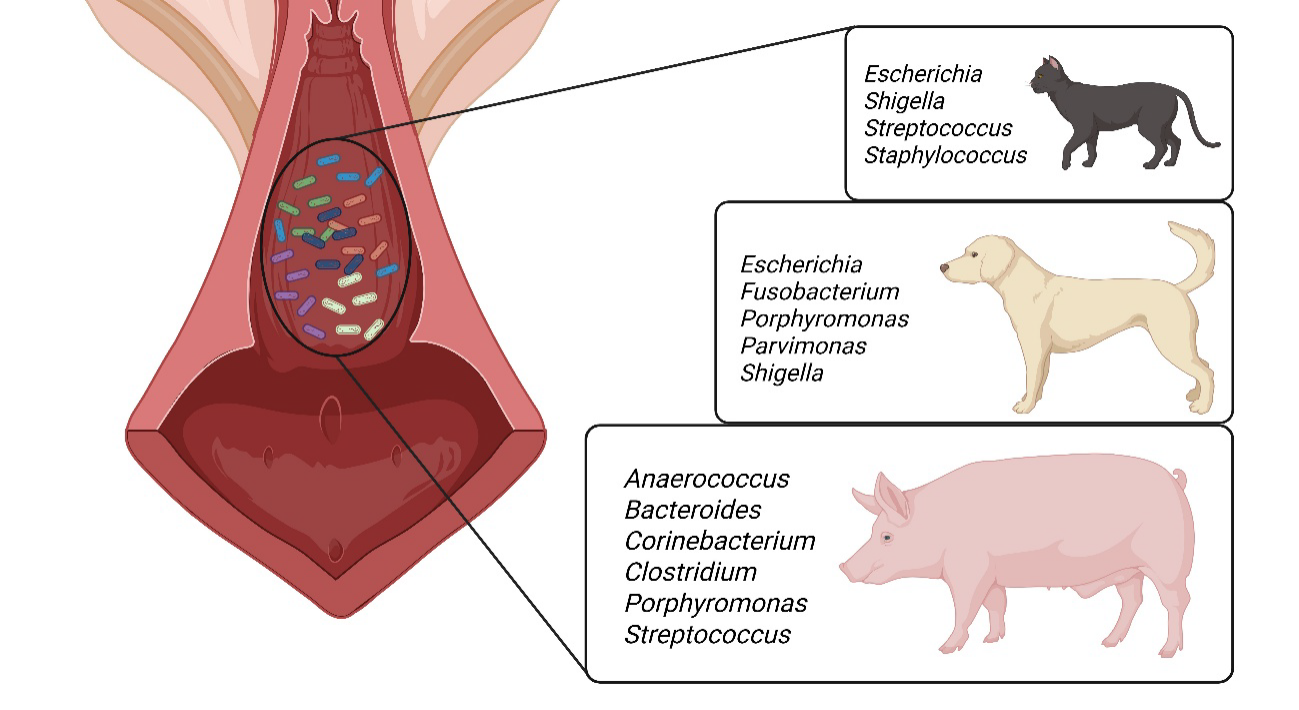
Overview of the most common bacterial genera identified via 16S rRNA sequencing in the vagina of healthy queens, bitches, and sows. Queens were either prepuberal or adult, in or out reproductive season, with no significant differences reported for alpha and beta diversity between groups. Bitches (5-9 yo) were sampled at 4 different phases of the oestrous cycle (proestrus, estrus, diestrus and anoestrus), with no significant differences reported for alpha diversity (richness and evenness) among phases. No additional information about age, pregnancy status or moment of the oestrus cycle was available for sows. Adapted from Liang et al. (2022), Banchi et al. (2024) and Gronsfeld et al. (2024).

The microbiota composition varies markedly across different body sites, with the reproductive tract harbouring distinct microbial niches such as the vagina, cervix, uterus, oviducts, and ovaries. Each organ’s microenvironment fosters unique microbial communities, yet their composition and activity in a healthy state can shift due to factors like pH, oxygen levels, hormonal fluctuations, temperature, metabolites, circadian rhythms, and other physiological dynamics. Species-specific traits further shape these niches (Figures 1, 2 and 3), highlighting the adaptability of microbial communities to internal and external influences. For instance, the cervix, which serves as a bridge (and a gate) between the vagina and uterus, is periodically opening during oestrus, potentially modifying the uterine environment by increasing oxygen levels. Moreover, elevated oestrogen during oestrus triggers hyperaemia, mucus production, and immune cell migration into the uterine lumen, which may help prevent pathogenic bacteria from ascending from the cervix and vagina. Semen deposition during natural or artificial insemination introduces additional microorganisms, further influencing the reproductive tract microbiome. In the luteal phase, the cervix closes, oxygen levels drop, uterine hyperaemia subsides, immune activity decreases, and the microbiome stabilizes.

In species like cows (with synepitheliochorial placentation) and dogs (with endotheliochorial placentation), blood accumulates in the uterine lumen postpartum, potentially introducing hematogenous microbes, often gut-derived, into the uterine microbiome. This environment, rich in decomposing material, can promote the growth of commensal yet potentially pathogenic bacteria. Effective immune regulation and uterine ecbolic competence are crucial for maintaining uterine health at this stage. In species with less invasive placentation (e.g., mares and sows), ascending vaginal microbes primarily shape the uterine microbiome, with local immune modulation playing a key role in maintaining homeostasis.

Most research on reproductive tract in domestic animals has concentrated on the vaginal and uterine microbiomes, leaving the cervix, oviduct, and ovaries relatively underexplored. The following sections review in detail the origin, composition, and dynamics of the healthy reproductive microbiome in domestic animals.

### Dynamics of the healthy reproductive tract microbiome in the cow

Moore et al. (2017) were the first to identify 16S rRNA gene sequences in the uteri of virgin heifers around oestrus, with Firmicutes, Bacteroidetes, and Proteobacteria dominating at relative abundances of 40, 35, and 10%, respectively. Recently, Quereda et al. (2020) analysed vaginal microbiota changes in virgin heifers during the follicular (oestrus) and luteal (day 14 post-oestrus) phases. They detected slightly greater diversity in the follicular phase, with 27 bacterial phyla, compared to 23 phyla in the luteal phase. While β diversity analysis revealed differences between the two phases, taxonomic variations were limited to low-abundance taxa, which were often absent in some samples. Similar to the findings of Moore et al. (2017) in the virgin uterus, Tenericutes, Firmicutes, and Bacteroidetes consistently accounted for over 75% of the vaginal microbiota (Quereda et al., 2020). Thirty-two genera exhibited fluctuations in relative abundance across the cycle. Notably, *Lactobacillus* was present at low levels (<1%) throughout but was more abundant in the follicular phase than in the luteal phase. Moraes et al. (2024a) explored the colonization of heifer uterine microbiome after insemination with sterile PBS to test the hypothesis that this procedure contributes to contamination of the uterus by microbes derived from vagina or cervix. They concluded that insemination did not significantly alter the uterine microbiome composition, as measured by bacterial culture and 16S rRNA gene sequencing. The bacteria detected in the uterus of virgin heifers were typical of those found in the soil, environment, skin, mucous membranes, and urogenital tract of animals, including genera such as *Bacillus*, *Corynebacterium*, *Cutibacterium*, *Micrococcus*, *Staphylococcus*, and *Streptococcus*. These findings suggest that the uterine microbiome in virgin heifers is likely influenced by environmental and host-associated sources, following a potential route of transfer: environment → skin → vagina → uterus. A summary of the most common bacterial phyla found in the vagina and uterus of non-pregnant, cycling heifers is shown in Figures 1 and 2.

The presence of a microbiota in the bovine pregnant uterus was first described by Karstrup et al. (2017) and later confirmed by Moore et al. (2017). The first one identified *Fusobacterium necrophorum*, *Porphyromonas levii*, and *Trueperella pyogenes* within the endometrium samples collected post-mortem from the pregnant uteri of cows using fluorescence *in situ* hybridization. Parallel 16S rRNA sequencing revealed that the most abundant bacterial families were Porphyromonadaceae, followed by Ruminococcaceae and Lachnospiraceae. Similarly, Moore et al., (2017) collected samples from cows in the last trimester of pregnancy (e.g., amniotic fluid, inter-cotyledonary placenta, placentome tissue, and cervical lumen) and found that Firmicutes, Bacteroidetes, and Proteobacteria were the most abundant phyla, accounting for 35-42%, 23-33%, and 2-15% of the reads, respectively. Both studies reported low but detectable biomass, indicating the presence of a microbial community in the pregnant uterus. More recently, Moraes et al. (2024b) revisited this model, conducting both bacterial culture and 16S rRNA gene sequencing in parallel. They included samples from the external surface of the pregnant uterus for comparison. Samples derived from within the pregnant uterus consisted of placentome, inter-cotyledonary placenta, inter-caruncular endometrium, amniotic fluid, allantoic fluid, foetal abomasum content, and foetal meconium. Notably, *Lactococcus lactis*, *Macrococcus flavus*, *Staphylococcus hominis*, and *Streptococcus sanguinis* were isolated from the internal uterine environment but not from external samples. No distinct microbiome signature was identified across different regions of the pregnant uterus. However, genera such as *Peptoniphilus*, *Actinomyces*, *Finegoldia*, and *Haemophilus* were found in relatively high prevalence within the uterus but were either absent or present at low levels on the external surface. The authors concluded that the pregnant uterine microbiome represents a low-biomass environment with very few viable (culturable) bacteria (Moraes et al., 2024b). These findings raise an important question: What role, if any, does this low-biomass microbiome play in the physiology of a normal, healthy bovine pregnancy? Figure 1 shows the most common bacterial phyla found in the uterus of pregnant cows.

In the early postpartum period, the compartmentalization between the vagina and uterus breaks down, leading to a significant increase in microbial biomass within the genital tract (Bicalho et al., 2017a). Traditionally, it was believed that bacteria in the postpartum uterus ascended from the vagina to colonize the uterine cavity (Çömlekcioğlu et al., 2024). However, recent evidence suggests that hematogenous transmission may also play a role in shaping the postpartum uterine microbiome. This hypothesis is supported by the observation that *Fusobacterium necrophorum* and *Trueperella pyogenes*, known to travel from the rumen to the liver via the bloodstream causing liver abscesses, might similarly reach the uterus through blood circulation. Jeon et al. (2017) tested this idea and identified major uterine pathogens, including *Bacteroides*, *Porphyromonas*, and *Fusobacterium*, as part of the core bacterial genera in blood samples collected from cows on the day of calving and two days postpartum. Since maternal blood enters the uterus after calving, free-floating bacteria can migrate into the endometrium, suggesting that hematogenous transmission may be a significant route for at least some uterine pathogens.

The postpartum vagina and uterus are high biomass environments. The dominant bacterial phyla in these organs include Proteobacteria, Firmicutes, Bacteroidetes, Fusobacteria, Tenericutes, and Actinobacteria (Bicalho et al., 2017a, b). However, there is no consensus on the core microbial composition of the healthy bovine genital tract, as uterine microbial populations vary widely in diversity and relative abundance among individuals (Çömlekcioğlu et al., 2024). Factors such as the stage of the oestrous cycle, time relative to parturition, parity, breed, and genetics influence these microbial dynamics. Additionally, herd management factors like nutrition, dystocia, and housing conditions also impact the reproductive tract microbiome. Discrepancies in microbial diversity reported across studies may further arise from differences in methodological approaches (Lietaer et al., 2021, 2023). A simplified overview of bacterial phyla found in the vagina and uterus of healthy postpartum cows is shown in Figures 1 and 2.

In mid-lactation dairy cows, the functional cervix reestablishes compartmentalization between the vagina and uterus, resulting in distinct microbiome compositions in each environment (Miranda-CasoLuengo et al., 2019; Lietaer et al., 2021). The vaginal biomass is typically three to four orders of magnitude greater than that of the uterus, with both considered low-biomass environments (Lietaer et al., 2021). A study incorporating appropriate controls found that uterine samples had higher abundances of Pseudomonadaceae and Burkholderiaceae but lower abundances of Pasteurellaceae, Ruminococcaceae, Mycoplasmataceae, Rikenellaceae, Corynebacteriaceae, and Lachnospiraceae compared to vaginal samples (Lietaer et al., 2021). Interestingly, no significant differences in bacterial community structure (α and β diversities) were observed between different regions of the uterus, such as the tip and base of the left and right uterine horns.

To date, no peer-reviewed studies have characterized the composition of the oviductal or ovarian (follicular) microbiome in cows. We hypothesize that the oviductal microbiome resembles the uterine microbiome, both in health and disease. The most probable route of bacterial colonization in the oviducts is ascending from the uterus, though an alternative pathway through the abdominal cavity (via the infundibulum) cannot be ruled out. In humans, a follicular fluid microbiome has been documented, but in cows, this area remains entirely unexplored (Pelzer et al., 2013). Notably, the cow exhibits a unique anatomical relationship between the ovarian arteries and uterine veins, which is essential for the local transfer of prostaglandins from the endometrium to the ovary (Knickerbocker et al., 1988). We propose that this specialized vascular arrangement may not only facilitate the transfer of prostaglandins and inflammatory or bacterial byproducts to the ovaries (Gilbert, 2011), but could also serve as a potential route for bacterial translocation. This mechanism might enable bacteria to colonize the ovary, potentially leading to the establishment of a follicular microbiome. Further research is needed to investigate this hypothesis and elucidate the role of microbial communities in ovarian physiology and pathology.

### Dynamics of the healthy reproductive tract microbiome in the mare

The vaginal microbiome of horses has been reported to consist predominantly of bacteria, with archaea detected at minimal abundances (Płoneczka-Janeczko et al., 2024). The core vaginal microbiome in healthy mares is primarily composed of the phyla Firmicutes, Bacteroidetes, Proteobacteria, and Actinobacteria (Barba et al., 2020; Malaluang et al., 2024) (Figure 1) At the genus level, dominant taxa include *Porphyromonas*, *Campylobacter*, *Arcanobacterium*, *Corynebacterium*, *Streptococcus*, and *Fusobacterium* (Barba et al., 2020). However, the relative abundance of these phyla and genera varies significantly across studies, and no consensus exists regarding fluctuations in microbiome composition during the oestrous cycle. For instance, Barba et al. (2020) found no differences in microbial communities between mares in oestrus and dioestrus. In contrast, Malaluang et al. (2024) observed dynamic associations between bacterial species and sampling days, suggesting cyclical variability. Notably, methodological differences may contribute to these discrepancies: while both studies used swab-based sampling of the caudal vagina, their sampling timelines differed. Barba et al. (2020) compared two phases (oestrus and dioestrus, determined via reproductive tract examination and hormone levels), whereas Malaluang et al. (2024) analysed four time points (ovulation [day 0], day 3, day 7, and days 14) to account for hormonal fluctuations.

Further research by Heil et al. (2024) demonstrated that the oestrous cycle influences uterine microbial communities. While mares in oestrus and anoestrus showed similar phyla-level composition, distinct genera predominated in each phase. During anoestrus, the uterine microbiota exhibited greater diversity and richness, with Rikenellaceae RC9 gut group and Peptoanaerobacter as dominant taxa. In contrast, *Klebsiella*, *Aeromonas*, *Mycoplasma*, and *Citrobacter* were more abundant during oestrus. These findings suggest that oestrogen exposure may alter the uterine environment, favouring specific microbial communities and potentially impacting fertility outcomes. Methodologically, Heil et al. (2023) found that sampling technique—whether swab, low-volume lavage, or biopsy—did not significantly affect microbiome profiling results.

Holyoak et al. (2022) characterized the microbiome of the healthy equine uterus, identifying the phyla Proteobacteria, Firmicutes, Bacteroidetes, Actinobacteria, and Kiritimatiellaeota (Figure 2), along with the genera *Lactobacillus*, *Escherichia*/*Shigella*, *Streptococcus*, *Blautia*, *Staphylococcus*, *Klebsiella*, *Acinetobacter*, and *Peptoanaerobacter*, which were consistently present across the 54 samples they collected. Their study also revealed significant geographical variation in uterine microbiota composition, with differences in species diversity, richness, and evenness among mares from Oklahoma, Louisiana, Australia, and the Southern Mid-Western United States.

Regarding the placental microbiome in mares, van Heule et al. (2023) characterized the microbial population in the equine placenta (chorioallantois) of healthy mares, both prepartum (280 days of gestation, n = 6) and postpartum (immediately after foaling, 351 days of gestation, n = 11), using 16S rDNA sequencing. In both groups, the dominant bacteria belonged to the phyla Proteobacteria, Firmicutes, Actinobacteria, and Bacteroidota. The five most abundant genera identified were *Bradyrhizobium*, an unclassified *Pseudonocardiaceae*, *Acinetobacter*, *Pantoea*, and an unclassified *Microbacteriaceae*. Alpha diversity and beta diversities were different between the pre- and postpartum samples. Additionally, the abundance of 7 phyla and 55 genera varied significantly between the two groups. These findings suggest that the caudal reproductive tract microbiome influences (contaminates) the postpartum placental microbial composition, with the passage of the placenta through the cervix and vagina during parturition having a notable impact. This study, for the first time, provides compelling evidence of bacterial DNA in healthy equine placentas and sets the stage for further exploration into the role of the placental microbiome in the genesis of placentitis and pregnancy development. Bacterial phyla found in the uterus (placenta) of preterm pregnant (280 days of gestation) mares are depicted in Figure 2.

No publicly available data currently exist on the follicular or oviductal microbiome in mares. Given the insights gained from human reproductive studies (and the critical role of optimal fertility in the equine industry) future research should prioritize characterizing the follicular microbiome, its impact on fertility outcomes, and the factors shaping its composition. Similarly, while the oviductal microbiome remains poorly understood across species, investigating this niche could unlock vital insights into fertilization success and early embryonic development. Addressing these gaps could revolutionize reproductive management strategies in equine medicine.

### Dynamics of the healthy reproductive tract microbiome in the sow, bitch, and queen

Compared to cows and mares, research characterizing the composition and dynamics of the healthy reproductive microbiome in sows, bitches, and queens remains limited. Existing studies often involve small sample sizes and are further complicated by substantial breed variations (specially in dogs), which may introduce bias and hinder generalizability.

A summary of the most prevalent bacterial genera identified in the vagina of healthy sows, bitches, and queens is shown in Figure 3. In healthy sows, the predominant vaginal genera include *Corynebacterium_1*, *Clostridium_sensu_stricto_1*, *Porphyromonas*, *Anaerococcus*, *Streptococcus*, and *Bacteroides* (Liang et al., 2022). In bitches, the most abundant vaginal genera are *Fusobacterium*, *Porphyromonas*, *Parvimonas*, and *Escherichia-Shigella* (Gronsfeld et al., 2024). In queens, Banchi et al. (2024) identified mixed and monoculture communities of *Escherichia coli*, *Streptococcus canis*, *Staphylococcus felis*, and *Enterococcus spp*. as normal components of the healthy vaginal microbiome. The vaginal microbiota composition in bitches can be influenced by the oestrous cycle phase (Lyman et al., 2019; Golińska et al., 2021), though no significant uterine microbiota changes were observed across cycle stages (Golińska et al., 2021). Sterilization may also alter the reproductive microbiome in bitches, though findings were limited by small sample sizes and animals sterilized after sexual maturity (Rota et al., 2020). Interestingly, the vaginal microbiota of queens remains unaffected by age or season (Banchi et al., 2024).

In sows, dietary interventions such as late-gestation lysozyme supplementation may modulate vaginal microbiota and enhance reproductive efficiency (Xu et al., 2021). Diet-induced shifts in the faecal microbiome can indirectly influence reproductive tract microbiomes. Environmental conditions further shape microbial communities, particularly in pigs, where suboptimal management increases susceptibility to stress, reproductive infections, and reduced productivity (Einarsson et al., 2008). Vaginal microbiome variations across farms underscore the impact of farming practices in sows (Liang et al., 2022). Furthermore, artificial insemination may destabilize the sow uterine microbiome due to their large ejaculate volumes, particularly when uterine clearance mechanisms are impaired, raising risks of post-breeding induced endometritis (PBIE, Pascottini et al., 2023a).

A small-scale study on bitches (n = 5) and queens (n = 3) undergoing elective caesarean sections analysed endometrial, amniotic fluid, meconium, and environmental control samples via culture and 16S rRNA sequencing (Banchi et al., 2023). Culture resulted in growth of common, non-specific bacteria and sequencing revealed lower bacterial abundance in feto-maternal tissues compared to controls. Species-specific microbial profiles emerged, with taxonomic differences between canines and felines at the order, family, and genus levels. Dominant phyla included Bacteroidetes, Firmicutes, and Proteobacteria, varying by tissue and species. Alpha and β diversities did not differ between feto-maternal tissues and controls. These results suggest low bacterial biomass in healthy term pregnancies of dogs and cats, likely originating from maternal skin contamination rather than a resident (viable) community.

## Loss of microbial diversity: a risk for reproductive tract inflammatory diseases

The female reproductive tract hosts a diverse community of commensal microorganisms that play a crucial role in pathogen control through competitive exclusion and immune system priming. However, when microbial balance is disrupted, disease can arise. Before parturition, anatomical barriers such as the vulvar lips, vestibule-vaginal junction, and cervix regulate microbial exchange between the uterus and the vagina. However, postpartum, these barriers become compromised, leading to a loss of compartmentalization within the reproductive tract.

Postpartum uterine inflammation is a necessary physiological response across domestic species. It facilitates immune cell recruitment, particularly polymorphonuclear leukocytes (PMN) and macrophages, which helps regulate bacterial proliferation and prevent uterine dysbiosis. During this period, the uterus expels debris through lochia discharge, modulates inflammation, and undergoes endometrial remodelling to achieve uterine involution. However, factors such as prolonged or difficult labour, unskilled obstetric intervention, and retained foetal membranes significantly increase the risk of bacterial contamination and subsequent uterine disease. Metritis and endometritis are among the most common postpartum uterine disorders in domestic animals. While metritis is a systemic condition that can become life-threatening due to widespread infection and systemic illness, endometritis is typically localized to the uterus but may extend to nearby structures, including the vagina, oviducts, and ovaries. Although not life-threatening, endometritis can have lasting effects on fertility, both in the short and long term.

In mares, sows, and bitches, a substantial volume of ejaculate is deposited directly into the uterus during mating. Under normal conditions, uterine contractions and immune responses rapidly restore microbial homeostasis, causing transient post-mating endometritis, which resolves within hours to days. However, if uterine clearance mechanisms fail or immune function is insufficient (or exacerbated), PBIE can develop, disrupting the uterine microbiome and impairing fertility. Additionally, in mares and bitches, microbial infiltration into the uterus can occur independently of mating (during oestrus), particularly in individuals with poor perineal conformation, leading to post-oestrus endometritis.

### Reproductive tract inflammatory diseases in the cow

In dairy cows, the physiological postpartum uterine inflammation is challenged by factors such as high milk production and calving in open environments, leading to metabolic stress, systemic inflammation, compromised immunity, and increased uterine bacterial contamination during the early postpartum period (Pascottini and LeBlanc, 2020; Pascottini et al., 2023b). If not effectively managed, these challenges can result in RTID, affecting up to 50% of dairy cows after calving.

Within the first 21 days postpartum, particularly between days 3 and 10, cows are susceptible to developing metritis (Sheldon, 2019). This condition involves inflammation of all uterine layers—endometrium, myometrium, and perimetrium—and is clinically identified by a foul-smelling uterine discharge, accompanied by an enlarged and flaccid uterus.

Endometrium, the innermost lining of the uterus, comprises two layers: the superficial stratum compactum and the deeper stratum spongiosum. Endometritis refers to inflammation confined to the stratum compactum, leading to damage of the luminal epithelium, vascular congestion, swelling, and infiltration of inflammatory cells, primarily PMN. While postpartum endometritis is part of the normal tissue repair process and aids in clearing debris (lochia), delayed uterine clearance—evidenced by purulent vaginal discharge (PVD) after 21 days postpartum—is associated with reduced reproductive performance (Dubuc et al., 2010). Purulent vaginal discharge indicates the presence of pus in the vagina but does not always signify endometritis; it can also result from cervicitis, vaginitis, or a combination of these conditions (Dubuc et al., 2010; Deguillaume et al., 2012). Clinical endometritis (CE) is characterized by PVD accompanied by endometrial inflammation, often diagnosed via endometrial cytology (Dubuc et al., 2010). Subclinical endometritis (SCE), on the other hand, lacks visible signs but still adversely affects fertility (Wagener et al., 2017). Together, metritis, PVD, CE, and SCE constitute the RTID complex in dairy cows. These conditions vary in severity and prevalence but collectively impact reproductive health and fertility, posing significant challenges to dairy herd management and profitability.

Culture-dependent studies have established a strong correlation between metritis, PVD and CE with the presence of pathogenic bacteria, including *Trueperella pyogenes*, *Escherichia coli*, *Prevotella melaninogenica*, and *Fusobacterium necrophorum*, in samples collected from the genital tract of postpartum dairy cows (Table 1) (Williams et al., 2005; Carneiro et al., 2016; Gilbert and Santos, 2016). Advancements in culture-independent techniques have further identified genera such as *Bacteroides*, *Fusobacterium*, *Helcococcus*, *Filifactor*, and *Porphyromonas* as being associated with metritis, and *Bacteroides*, *Ureaplasma*, *Helcococcus*, *Fusobacterium*, *Trueperella*, *Prevotella*, and *Porphyromonas* with PVD and CE (Table 1) (Machado et al., 2012b; Miranda-CasoLuengo et al., 2019; Pascottini et al., 2020).

**Table 1:** Summary of the most common bacteria identified in the reproductive tract of females with reproductive tract inflammatory disease according to the diagnostic method.

**Species**	**Diagnostic method**	**Disease**	**Phylum**	**Genus**	**Species**	**References**
**Cow**	Culture-dependent	MET, PVD, CE	*Actinomycetota*	*Trueperella*	*Trueperella pyogenes*	Williams et al. (2005)
			*Pseudomonadota*	*Escherichia*	*Escherichia coli*	Carneiro et al. (2016)
			*Bacteroidota*	*Prevotella*	*Prevotella melaninogenica*	Gilbert and Santos (2016)
			*Fusobacteriota*	*Fusobacterium*	*Fusobacterium necrophorum*	
**Cow**	Culture-independent	MET	*Bacteroidota*	*Bacteroides*	*Fusobacterium necrophorum*	Machado et al. (2012b)
			*Fusobacteriota*	*Fusobacterium*	*Bacteroides pyogenes*	Jeon et al. (2016)
			*Firmicutes*	*Helcococcus*		Miranda-Casoluengo et al. (2019)
				*Filifactor*		Pascottini et al. (2020)
				*Porphyromonas*		Rashid et al. (2025)
		PVD, CE	*Bacteroidota*	*Bacteroides*	*Trueperella pyogenes*	
			*Firmicutes*	*Ureaplasma*	*Filifactor alocis*	
			*Fusobacteriota*	*Helcococcus*	*Paptoniphilus grossensis*	
			*Actinomycetota*	*Fusobacterium*	*Peptoniphilus obesi*	
				*Trueperella*		
				*Prevotella*		
				*Porphyromonas*		
				*Peptoniphilus*		
				*Peptostreptococcus*		
				*Helcococcus*		
		SCE	*Firmicutes*	*Anaerococcus*	*Aerococcus viridans*	Wang et al. (2018)
			*Actinomycetota*	*Corynebacterium*		Pascottini et al. (2020)
			*Pseudomonadota*	*Staphylococcus*		
				*Actinobacter*		
				*Lactobacillus*		
**Mare**	Culture-dependent	CEM	*Pseudomonadota*	*Taylorella*	*Taylorella equigenitalis*	Timoney and Powell (1988)
		END	*Pseudomonadota*	*Escherichia*	*Escherichia coli*	Liu and Troedsson (2008)
			*Firmicutes*	*Klebsiella*	*Klebsiella pneumoniae*	Paccamonti and Pycock (2009)
				*Pseudomonas*	*Pseudomonas aeruginosa*	
				*Streptococcus*		
	Culture-independent	END	*Pseudomonadota*	*Escherichia*	*Escherichia coli*	Virendra et al. (2024)
			*Proteobacteria*	*Salmonella*	*Salmonella enterica*	
				*Klebsiella*	*Klebsiella pneumoniae*	
**Sow**	Culture-dependent	PVD	*Actinomycetota*	*Trueperella*	*Trueperella pyogenes*	Poor et al. (2022)
			*Bacteroidota*	*Bacteroides*	*Bacteroides pyogenes*	
			*Firmicutes*	*Corynebacterium*	*Corynebacterium diphtheriae*	
				*Streptococcus*	*Streptococcus dysgalactiae*	
				*Staphylococcus*	*Staphylococcus hyicus*	
	Culture-independent	PVD	*Bacteroidota*	*Bacteroides*	*Bacteroides pyogenes*	
			*Firmicutes*	*Streptococcus*	*Streptococcus dysgalactiae*	
				*Porphyromonas*		
**Bitch**	Culture-dependent	CEH-	*Pseudomonadota*	*Escherichia*	*Escherichia coli*	Coggan et al. (2008)
		pyometra	*Firmicutes*	*Streptococcus*		Ylhäinen et al. (2025)
				*Staphylococcus*		
	Culture-independent	CEH-	*Mycoplasmatota*	*Mycoplasma*	*Pseudomonas aeruginosa*	Zheng et al. (2023)
		pyometra	*Firmicutes*	*Enterococcus*		
			*Pseudomonadota*	*Haemophilus*		
				*Pseudomonas*		

MET: metritis; PVD: purulent vaginal discharge; CE: clinical endometritis; SCE: subclinical endometritis; END: endometritis; CEM: contagious equine metritis; CEH: cystic endometrial hyperplasia.

Salpingitis is characterized by infection and inflammation of the oviducts. This condition can manifest as acute or chronic and may range in severity from mild to severe, typically resulting from infections ascending from the uterus. Histologically, salpingitis is marked by increased vascularization, heightened secretion, and notable infiltration of the oviductal tissues with PMN and plasma cells. Microbiological analyses of simultaneously-collected uterine and oviductal fluids from cows with varying degrees of inflammation have revealed that the same bacterial species are present in both the uterus and oviduct. In cases of salpingitis, *Trueperella pyogenes* has been identified through aerobic culture, but *Streptococcus pluranimalium* and *Fusobacterium necrophorum* have been detected in severe cases of salpingitis (Filatova et al., 2021; Sadeghi et al., 2022).

Inflammation of the ovaries and surrounding structures is termed oophoritis and perioophoritis, respectively. In bovines, perioophoritis is the more common ovarian pathology, while oophoritis appears to be rare. The aetiology of ovarian inflammation often stems from ovarian manipulations, infections originating from the uterus, or systemic diseases. Involvement of the mesosalpinx or salpinx in the inflammatory process exacerbates the condition. Trauma from improper palpation, enucleation of the corpus luteum, or manual rupture of cystic ovaries are common causes of oophoritis. Repeated or unskilled ovum pick-up procedures which facilitate bacterial contamination from the anterior vagina into the follicles can also lead to oophoritis and adhesions, with suppurative oophoritis and ovarian abscesses arising as potential complications. No study has attempted to evaluate the composition of the ovarian microbiome in cases of oophoritis or peri-oophoritis, though bacteria present in the diseased vagina, uterus, or oviduct are likely implicated.

### Reproductive tract inflammatory diseases in the mare

Endometritis is the leading cause of infertility and reduced reproductive performance in mares (Canisso et al., 2020). This condition is classified into three categories: acute infectious endometritis, PBIE, and chronic infectious endometritis (LeBlanc, 2010). The sequence of events driving the development of endometritis may follow this chronology: acute endometritis can occur postpartum due to delayed uterine involution, with retained foetal membranes (failure to expel the placenta within 6-8 hours of parturition) being the most important cause (Hurtgen, 2006; LeBlanc, 2008). Following breeding (whether by natural or artificial insemination), there is a transient, physiological post-breeding endometrial inflammation that typically resolves within 24 to 36 h (Katila, 1995). After this period, resistant mares efficiently eliminate exogenous agents (e.g., seminal plasma, microorganisms, uterine debris), whereas susceptible mares fail to clear these stimuli, progressing to PBIE (Troedsson, 1997). Persistence of endometrial inflammation, characterized by free fluid in the uterine lumen and an influx of PMN >36 hours post-breeding, is referred to as PBIE. Risk factors for PBIE include conformational abnormalities, such as pneumovagina, a pendulous uterus, or urine pooling, which delay uterine clearance and promote intrauterine fluid accumulation (Reilas et al., 1997; LeBlanc et al., 1998). Additional risk factors include impaired myometrial contractility and incomplete cervical dilation, often resulting from repeated foaling, aggressive reproductive manipulations, or fibrosis (Troedsson, 2008; LeBlanc and Causey, 2009). These conditions are not only associated with PBIE but also with continuous re-infection, which causes the condition to evolve from acute to chronic endometritis, often linked with persistent microbial colonization and residual tissue damage (Hurtgen, 2006).

Equine endometrosis is a chronic, progressive, and irreversible fibrosis affecting the endometrium (Rebordão et al., 2014). Endometritis and endometrosis are interconnected conditions that reinforce each other in a continuous cycle: when endometritis dominates, the uterine environment shifts from an inflammatory state to a fibrotic endometrium, increasing susceptibility to chronic endometritis and creating a hostile setting for sperm and early embryos, ultimately leading to infertility (Katila and Ferreira-Dias, 2022). Lastly, contagious equine metritis (CEM) is a venereal disease caused by the bacterium *Taylorella equigenitalis* (Timoney and Powell, 1988). Mating with stallions whose external genitalia are colonized by *Taylorella equigenitalis* (or their contaminated semen) often results in CEM, as does insemination with contaminated semen.

Culture-dependent methods have identified both acute and chronic endometritis, as well as PBIE, as associated with bacterial pathogens such as *Escherichia coli*, *Streptococcus* spp., *Staphylococcus* spp., *Klebsiella pneumoniae*, and *Pseudomonas aeruginosa* (Table 1) (Liu and Troedsson, 2008; Paccamonti and Pycock, 2009). More recently, 16S rRNA sequencing was used to compare samples collected from the uterus of 30 mares (Virendra et al., 2024), classified into healthy (n = 15) and (acute or chronic) endometritis (n = 15). In healthy mares, the most abundant phylum, class, order, and family were Firmicutes, Bacilli, Bacillales, and *Paenibacillaceae*, respectively. In contrast, the most abundant corresponding taxonomic levels in mares with endometritis were Proteobacteria, Gammaproteobacteria, Enterobacterales, and *Enterobacteriaceae*, respectively. At the genus level, *Brevibacillus* and *Paenibacillus* were more abundant in healthy mares, while *Escherichia*, *Salmonella*, and *Klebsiella* were more abundant in mares with endometritis (Table 1). A limitation of the study was that before sequencing, samples from mares diagnosed as healthy or with endometritis were randomly pooled, resulting in three and four sample batches being sequenced, respectively. This approach reduced statistical power and masked inherent individual variations, limiting the interpretation of the results. As for culture-independent methods to identify cases of mare endometritis, no further literature is currently available.

In mares, oviduct pathology is rarely recognized as a clinical factor contributing to infertility or during routine reproductive examinations (Schnobrich, 2019). However, a large study found that postmortem evaluations of the oviducts (both macroscopic and microscopic) revealed significant lesions in more than 85% of cases, including adhesions, cysts, fibrosis, and microscopic findings such as intraepithelial cysts, lymphocytic infiltration, and luminal proteinoid material (Saltiel et al., 1986). Mild multifocal subacute salpingitis is common in cases of CEM (Acland and Kenney, 1983), but aside from this, there are no studies linking bacteria in the context of oviduct pathology. Regarding the ovaries, ovum pick-up for the collection of immature oocytes for *in vitro* embryo production may lead to ovarian lesions, potentially resulting in ovarian abscesses. A recent report showed heavy growth of *Streptococcus equi* subsp. *zooepidemicus* from bacterial cultures of two ovarian abscess cases (Fernández‐Hernández et al., 2024). Therefore, special sanitary measures should be taken into account when repeatedly performing this procedure.

### Reproductive tract inflammatory diseases in the sow, bitch, and queen

In sows, postpartum metritis is characterized by uterine enlargement and the accumulation of intrauterine fluid (Sheldon et al., 2006). This condition is typically associated with severe clinical illness and is often linked to sepsis. While the incidence is relatively low, postpartum metritis can occur after prolonged labour, dystocia, retained foetal membranes, or in unsanitary farrowing conditions (Pascottini, et al., 2023a). Prolonged farrowing is also a major factor in the development of postpartum dysgalactia syndrome, also known as mastitis-metritis-agalactia (MMA, Kemper, 2020). The genesis of MMA is complex and usually involves the overgrowth of pathogenic microorganisms, such as *Escherichia coli*, *Klebsiella* spp., *Mycoplasma* spp., *Streptococcus* spp., and *Staphylococcus* spp., along with their endotoxins (Eckel and Ametaj, 2016). These endotoxins, especially from *Escherichia coli*, play a key role in the development of the condition (Marchant et al., 2000).

Literature on metritis in dogs and cats is limited. However, endometritis is the most significant postpartum condition in these species, as well as in sows (LeBlanc et al., 2002). The pathogenesis of endometritis is similar across species, with bacterial infections being the primary cause of the disease, although the specific pathogens may differ. *Escherichia coli* infections are more commonly reported in dogs and cats (Lawler et al., 1991; Fransson et al., 1997), while mixed infections (e.g., *Escherichia coli*, *Streptococcus* spp., *Staphylococcus* spp., *Klebsiella* spp., and *Mycoplasma* spp.) are more prevalent in sows.

Compared to cows and mares, diagnosing uterine pathologies in sows and companion animals is more challenging due to the lack of direct clinical examination methods. Access for *in vivo* uterine collection is difficult in these species, so cervical or vaginal swabs are often taken for bacteriological examination in suspected cases of endometritis. A recent large study used both culture-dependent (MALDI-TOF) and culture-independent (16S rRNA sequencing) methods to explore the microbiota of sows with purulent vaginal discharge (Poor et al., 2022). When compared to healthy, *Bacteroides pyogenes* was prominent in sows with vaginal discharge, while *Streptococcus dysgalactiae* and *Staphylococcus hyicus* were also found in higher relative abundance in affected sows (Table 1). Network analysis revealed important positive correlations between potentially pathogenic genera, such as *Escherichia-Shigella*, *Trueperella*, *Streptococcus*, *Corynebacterium*, and *Prevotella*, which were not present in healthy sows. In companion animals, to the best of our knowledge, no culture-independent study has evaluated the composition of the uterine or vaginal microbiota in bitches or queens with endometritis.

Cystic endometrial hyperplasia (CEH) is a frequently encountered condition in bitches, characterized by the abnormal hyperplasia and cystic dilation of the endometrial glands, often resulting from prolonged or excessive oestrogen exposure. This condition is more commonly observed in bitches than in queens and is particularly prevalent in older, unspayed females or those subjected to hormonal treatments aimed at oestrus suppression. Cystic endometrial hyperplasia leads to the accumulation of uterine fluid and thickening of the endometrial lining, creating an environment conducive to excessive bacterial growth. If untreated, CEH can progress to pyometra (De Bosschere et al., 2001; Verstegen et al., 2008). The most commonly implicated pathogens in pyometra using culture-dependent methods are *Escherichia coli*, *Streptococcus* spp., and *Staphylococcus* spp (summarized in Pascottini et al., 2023a). Amplicon sequencing revealed that, compared to healthy dogs, those with pyometra showed higher relative abundances of *Pseudomonas*, *Escherichia-Shigella*, *Mycoplasma*, *Enterococcus*, *Haemophilus*, *Vibrio*, and *Ralstonia*, while *Mycoplasma*, *Enterococcus*, and *Haemophilus* were comparatively less abundant in healthy controls (Zheng et al., 2023). Salpingitis and oophoritis are significantly understudied in companion animals, although two recent reports in bitches have provided detailed descriptions of these rare conditions (Hashemi et al., 2024; Nebel-Karp et al., 2025).

## Current therapies for reproductive tract inflammatory diseases

Therapeutic strategies for RTID vary across species, and are often shaped by legal regulations and economic factors. Regulatory authorities impose strict limitations on antimicrobial use in food-producing animals such as cows, mares, and sows (European Union, 2019a). The 'One Health' initiative, aiming to combat antimicrobial resistance, extends these considerations to companion animals like mares and bitches, encouraging the exploration of alternative treatments in these species as well. Additionally, legislation in several European countries (European Union, 2019b) has imposed more restrictive regulations on antibiotic use, not only limiting their application but also requiring antibiograms to justify the use of specific drugs, especially last-resort antibiotics.

In cows, local antimicrobial treatment following a precise diagnosis of CE and PVD is a viable option. Additionally, alternative non-antibiotic therapies are being explored, however, to date their outcomes are inconclusive. In mares, non-antibiotic agents are commonly used, but systemic antimicrobial treatment may be necessary depending on the severity of the condition. In sows, the prophylactic use of oxytocin and nonsteroidal anti-inflammatory drugs (NSAIDs) during the peripartum period has shown promise in reducing the incidence of postpartum endometritis. In bitches, treatment protocols are less defined, and therapy is typically administered based on clinical symptoms in affected animals.

### Therapies for reproductive tract inflammatory diseases in the cow

Metritis, PVD, and CE are reproductive disorders linked to dysbiosis, characterized by an increased relative abundance of pathogenic bacteria within the genital tract. In metritis, these bacteria and their toxins can enter the bloodstream, leading to systemic inflammation, fever, and septicaemia. In contrast, PVD and CE are localized conditions that do not cause systemic illness. Consequently, metritis involves parenteral antimicrobial treatment, whereas PVD and CE are typically managed with intrauterine therapy.

Studies indicate that antimicrobial treatment significantly improves metritis resolution rates. For example, clinical cure rates increase from approximately 60% in saline-treated controls to around 75% in cows receiving subcutaneous ceftiofur (Chenault et al., 2004). Regarding reproductive performance, cows with metritis treated with ampicillin or ceftiofur exhibited reproductive outcomes similar to healthy cows at first insemination (Lima et al., 2014). While this study lacked an untreated control group, it suggests that antimicrobial therapy mitigates the negative reproductive impact of metritis. Additionally, anti-inflammatory therapy has been explored to manage fever and improve feed intake in affected cows (Pohl et al., 2016; Paiano et al., 2024). However, current evidence supports its use only in combination with antimicrobial treatment, not as a standalone therapy.

For PVD and CE, intrauterine cephapirin administered at diagnosis has been associated with improved reproductive performance compared to no treatment (Denis-Robichaud and Dubuc, 2015). However, because these conditions are not life-threatening and antimicrobial resistance is a growing global concern, the routine use of antibiotics for PVD and CE remains controversial. Prostaglandin F2α (PGF2α) and its analogues have been investigated as alternative therapies to enhance uterine clearance and improve reproductive performance (Lefebvre and Stock, 2012). While early trials suggested potential benefits, they were criticized for unclear disease definitions and inadequate statistical power (LeBlanc et al., 2011). Subsequent meta-analyses found no significant improvement in healing or fertility outcomes following PGF2α treatment in cows with PVD or SCE (Lefebvre and Stock, 2012). Nevertheless, anecdotal evidence from veterinarians suggests that PGF2α may be beneficial in PVD or CE cases where a corpus luteum (CL) is present. The mechanism involves luteolysis, which reduces progesterone’s immunosuppressive effect, alongside enhanced uterine contractions that aid bacterial clearance. Additionally, oestrus induction increases uterine blood flow, promoting the recruitment of PMNs and immunoglobulins, which further support bacterial elimination. For SCE, a large, well-designed study demonstrated that intrauterine cephapirin administration at diagnosis improved first-service conception rates compared to untreated cows with SCE (Denis-Robichaud and Dubuc, 2015). This suggests that some SCE cases may involve mild bacterial infections or delayed uterine involution, similar to the recovery process observed in cows post-metritis or CE. As a result, these cases may benefit from targeted, local antimicrobial therapy.

Given the concerns over antimicrobial resistance, alternative, non-antibiotic therapies for RTID have been explored. Investigated treatments include intrauterine infusion of 50% dextrose, herbal extracts, ozone, and autologous serum (Heuwieser et al., 2000; Escandón et al., 2020; Eshghi et al., 2022; Paiano and Baruselli, 2022). However, many studies lacked proper randomization, statistical power, or double-blinding, limiting the reliability of their findings. While uterine lavage with saline may help flush out inflammatory cells, it remains impractical as a field treatment (Dini et al., 2015). Additionally, vaccination strategies targeting uterine pathogens have shown promise in experimental studies, with bacterin and toxoid vaccines administered to heifers in late pregnancy reducing metritis incidence (Machado and Silva, 2020). However, no commercial vaccine is currently available. Similarly, thorough research is being conducted on the role of antimicrobial peptides (AMPs) in reproductive health and their potential as alternative therapeutic agents. Antimicrobial peptides are small, cationic molecules that form part of the innate immune response (Li et al., 2018) that play a protective role in the reproductive tract (Subramanyam and Yeddula, 2024). It has been described that some of these molecules, such as cathelicidins, are implicated in the immune response during bovine endometritis (Li et al., 2021). Although promising results have been reported using AMPs to treat *Staphylococcus aureus*-induced endometritis in mice (Li et al., 2020), to date most studies in cattle have focused primarily on the gastrointestinal tract, and the broader implementation remains limited due to concerns regarding their *in-vivo* effectiveness, stability, and high cost of production (Rodrigues et al., 2022).

The use of bacteriophages constitutes another alternative to antibiotics. Some *in-vitro* studies reported the complete growth inhibition of *Escherichia coli* isolates from the uterus of postpartum dairy cows, including multidrug-resistant bacteria (Bicalho et al., 2010; Santos et al., 2010). However, *in-vivo* studies did not observe a beneficial effect of bacteriophage therapy for prevention of metritis and CE (Machado et al., 2012a; Meira et al., 2013). Consequently, additional clinical studies are needed to determine the true potential of bacteriophages, accounting for the main limitations such as host specificity and the selection of phage-resistant bacterial strains (Zduńczyk and Janowski, 2020).

Probiotics have gained attention as a sustainable approach to mitigate uterine diseases by modulating the vaginal and uterine microbiota and enhancing the host’s immune response (Wieërs et al., 2020). The rationale for employing probiotics in cattle originates from their successful application in addressing human reproductive infections, particularly bacterial vaginosis (Wang et al., 2019). In humans, the vaginal microbiota is predominantly composed of Lactobacilli, which inhibit pathogenic microorganisms by lowering vaginal pH through lactic acid production (Stojanović et al., 2012). Additional mechanisms include the synthesis of hydrogen peroxide, bacteriocins, and surface-binding proteins that prevent bacterial adhesion to uroepithelial cells (Stojanović et al., 2012). However, when compared to humans, the bovine vaginal microbiota exhibits a significantly lower relative abundance of Lactobacilli (Swartz et al., 2014). This disparity aligns with differences in vaginal pH between species: the bovine vaginal pH ranges from 5.5 to 8.6, whereas the healthy human vaginal pH is more acidic, typically between 3.8 and 4.5 (Beckwith-Cohen et al., 2012). Consequently, the suitability of lactic acid bacteria (LAB) as probiotics for modulating the genital tract microbiome in cattle remains uncertain. To date, no study has demonstrated successful colonization or sustained persistence of LAB in the bovine genital tract following treatment. Nevertheless, both *in vitro* models and field studies have shown promising results, with LAB enhancing immune responses and reducing the incidence of uterine diseases in dairy cows (Genís et al., 2016, 2017). However, many field studies have been limited by small sample sizes or lack of evaluation of uterine invasion and persistence of LAB post treatment, rendering their findings preliminary and necessitating cautious interpretation (Genís et al., 2018; Madureira et al., 2023).

### Therapies for reproductive tract inflammatory diseases in the mare

Effective treatment of RTID in mares aims to strengthen uterine defence, combat harmful bacteria, and manage inflammation. To achieve this, several approaches were considered, including surgical correction of anatomical abnormalities (such as the Caslick’s procedure), improving physical drainage post-insemination or breeding, regulating the inflammatory response, and local treatment aiming at preventing bacterial proliferation (LeBlanc, 2010).

For years, post-breeding inflammation has typically been managed by promoting the physical removal of uterine fluid, with uterine lavage and the use of ecbolic drugs (e.g., oxytocin) being the most common treatments (LeBlanc, 2010; Morris et al., 2020). In addition to these core mechanical strategies, therapies targeting the immune response, such as anti-inflammatory drugs, antibiotics, mucolytics, and immunomodulatory treatments, may also be used (Morris et al., 2020). However, it is important to recognize that post-breeding inflammation is a natural defence mechanism, and healthy mares can effectively evacuate uterine fluid on their own. Therefore, interventions should be reserved for mares that require assistance, and routine treatments should be avoided (Katila and Ferreira-Dias, 2022).

Often combined with uterine lavage, antibiotics are infused into the uterus or administered systemically after mating, with β-lactams and aminoglycosides being the most commonly used (Canisso et al., 2020). Although this approach is widely practiced by veterinarians, the administration of antimicrobials is most effective when bacterial contamination is identified as the cause of endometritis (Liu and Troedsson, 2008; Canisso et al., 2020). However, the irrational and routine use of antibiotics over recent decades has contributed to the development of antimicrobial resistance, posing a threat to both animal and human health. Common bacteria isolated from mares with endometritis, including *Streptococcus zooepidemicus* and *Escherichia coli*, have shown significant resistance to commonly used antimicrobials (Canisso et al., 2020). Additionally, antibiotic treatment failure in endometritis cases may result from biofilms produced by certain gram-negative bacteria, yeast, and fungi. Biofilms are complex microbial aggregates encased in an extracellular matrix, living in symbiosis, which protect the microorganisms from antibiotics (Walker, 2008; LeBlanc, 2010). Biofilms contribute to antibiotic resistance by limiting drug penetration reducing the concentration of the drug reaching bacterial cells. Furthermore, the density of target cells within a biofilm, a phenomenon known as the inoculum effect, can influence antibiotic susceptibility, limiting drug penetration and reducing the metabolic and growth rates of bacteria within them (LeBlanc, 2010). *Pseudomonas aeruginosa*, *Staphylococcus epidermidis*, *Escherichia coli*, and *Enterobacter cloacae*—bacteria involved in mare endometritis—are known to be strong biofilm producers (LeBlanc, 2010). To enhance the effectiveness of antimicrobials and disrupt biofilms, the addition of tris-EDTA or dimethyl sulfoxide (DMSO) has been shown to be beneficial (LeBlanc, 2010; Ferris et al., 2016; Loncar et al., 2017; Morris et al., 2020). Finally, although the mechanism is not yet fully understood, continuous and excessive use of antimicrobials has been linked to a higher incidence of fungal endometritis in mares (Liu and Troedsson, 2008). Treatment strategies for fungal endometritis have been discussed extensively elsewhere (Dascanio et al., 2001; Blanchard et al., 2003).

To address the growing issue of antimicrobial resistance, current research is focusing on alternative therapies to minimize the use of antibiotics. In the context of endometritis in mares, these therapies aim to regulate inflammation while also providing some antibacterial effects (Morris et al., 2020). The use of NSAIDs remains controversial, as they may reduce the production of PGF2α and impair myometrial contractions, which could delay uterine clearance (Canisso et al., 2020). In fact, delayed uterine clearance and increased inflammatory responses have been observed in mares treated with non-selective COX-2 NSAIDs (Canisso et al., 2020). An alternative approach involves the use of selective COX-2 NSAIDs, such as firocoxib or vedaprofen (Rojer and Aurich, 2010; Friso et al., 2019). Another potential therapy is chemical curettage, utilizing agents such as DMSO, hydrogen peroxide, magnesium sulfate, kerosene, and diluted disinfectants (Liu and Troedsson, 2008). Although research on the effectiveness of these agents is limited, it has been suggested that their success may be linked to the strong inflammatory response they trigger, which can stimulate myometrial contractions and enhance uterine clearance (Liu and Troedsson, 2008).

An alternative approach to treating endometritis in mares involves regenerative medicine, including the use of platelet-rich plasma (PRP) and stem cells (Del Prete et al., 2024). Intrauterine administration of PRP has been shown to regulate the uterine inflammatory response to semen by reducing the concentration of PMN, which leads to a reduction in endometrial thickness, oedema, and intrauterine fluid in mares (Reghini et al., 2016; Morris et al., 2020; Del Prete et al., 2024). Additionally, clinical trials have reported improved pregnancy rates in mares with reproductive challenges following PRP treatment (Metcalf, 2014). Moreover, recent studies suggested that fertility outcomes in mares treated with mesenchymal stem cells (MSCs) were significantly improved compared to those treated with PRP (Del Prete et al., 2024). Despite the promising results of regenerative medicine for treating endometritis, the number of clinical studies remains limited, and many of these studies have biases and variability in protocols. Therefore, further *in vitro* and *in vivo* research is required to optimize these therapies before they can be widely implemented in routine veterinary practice (Del Prete et al., 2024).

In mares, LAB such as *lactobacilli* and *enterococci*, are commonly used in commercial probiotic formulations (Lee et al., 1999). However, the reported beneficial effects vary across different isolates of vaginal lactic acid bacteria. Despite this variability, the results demonstrate promising potential for their use as equine probiotics (Fraga et al., 2008). More recently, five strains of *Enterococcus* spp. (*E. faecium* [two strains], *E. hirae* [two strains], and *E. gallinarum* [one strain]) were identified and selected for their beneficial properties in preventing urogenital infections in horses. This research paves the way for the development of a multi-strain probiotic formula aimed at preventing and treating equine endometritis (Silva et al., 2024).

### Therapies for reproductive tract inflammatory diseases in the sow, bitch, and queen

The primary treatment for RTID in sows and companion animals remains antibiotic therapy, despite concerns over antibiotic resistance and the potential risks to public health. In companion animals, the use of antibiotics is often influenced by pressure from breeders, which can contribute to overuse. Milani et al. (2012) reported that in some kennels, antibiotics are administered before and after birth to reduce puppy mortality. However, their study demonstrated that excessive antimicrobial use in breeding bitches during the peripartum period reduces the diversity of bacterial flora without decreasing the frequency of isolation of potentially pathogenic bacterial strains. Furthermore, pathogenic bacteria exhibit increased resistance to antibiotics, putting puppies at a higher risk of difficult-to-treat infections (Milani et al., 2012).

In the context of public health safety, domestic dogs have been identified as a potential source of multidrug-resistant *Escherichia coli* strains in households (Carvalho et al., 2016). In pigs, Wang et al. (2020) reported the isolation of *Streptococcus porcinus* in cases of endometritis. This bacterium is commonly associated with pyogenic infections, abortions, and endocarditis in pigs, as well as genitourinary tract infections in humans. The isolated strain exhibited multidrug resistance to aminoglycosides, quinolones, macrolides, and tetracyclines, while remaining sensitive only to certain β-lactams such as penicillin G, cephalothin, cefazolin, cephradine, and cefuroxime (Wang et al., 2020). The development of antibiotic resistance in pig farms represents a serious threat to human health, emphasizing the need for careful management of antibiotic therapy and the exploration of alternative therapeutic approaches.

Some research groups are actively working to develop treatment methods that are independent of antibiotics or are exploring alternative therapeutic substances in sows. Ye et al. (2021) proposed lysostaphin (a bacteriolytic enzyme that specifically targets and kills *Staphylococcus* species) as a potential alternative to antibiotics for treating endometritis in pigs. Intrauterine administration of lysostaphin significantly reduced the number of pathogenic bacteria, particularly *Staphylococcus aureus*, showing better results than oxytetracycline, highlighting its potential as a substitute for conventional antibiotics (Ye et al., 2021). The role of NSAID is of significant interest, particularly after farrowing. Studies have explored their effectiveness in addressing weak piglet syndrome and improving litter survival. Drugs like ketoprofen (Claeyé et al., 2015), meloxicam, and paracetamol (Plush et al., 2021) demonstrate anti-inflammatory effects that help reduce mucosal inflammation. However, there is a lack of dedicated publications on the impact of NSAIDs on uterine health, emphasizing the need for further research in this area.

It is important to note that the available information on the effectiveness and safety of various treatment methods in dogs, cats, and pigs is limited, with most studies focusing on individual cases or small animal groups, making it difficult to draw broad conclusions. Consequently, there is a pressing need for more extensive research to better understand disease mechanisms and develop optimal non-antibiotic therapeutic strategies for these species.

## From dysbiosis to balance: exploring the role of microbiome transplantation for managing reproductive tract inflammatory diseases in domestic animals

Since the pioneering studies on the gut microbiome and its role in obesity (Sonnenburg et al., 2004; Bäckhed et al., 2005), microbiome research has evolved significantly. The launch of the NIH Human Microbiome Project (NIH, 2025b) marked the first large-scale effort to map the human microbiome, shifting the field from simply cataloguing microbial communities to understanding the complex principles that regulate their structure, function, and dynamics. This deeper insight into host-microbiome interactions and microbial ecosystems has paved the way for advanced microbiome-based therapies aimed at restoring microbial balance and enhancing host health. One of the most promising approaches is microbiome transplantation—transferring a healthy donor's microbiome to a recipient to improve health outcomes, treat disease, or prevent illness. By leveraging the essential role of microorganisms in maintaining physiological homeostasis, microbiome transplantation represents a powerful tool for addressing microbiome-associated disorders across various species.

Currently, the use of microbiome transplantation to prevent and treat RTID in domestic species is virtually non-existent. However, much can be learned from its application in other species, such as humans and mice. The role of microbiome transplantation in restoring balance and diversity has been extensively studied for treating microbiome-associated diseases in the human gut. As a result, faecal microbiome transplantation (FMT) has become the most established and widely used microbiome therapy, primarily for treating *Clostridium difficile* infections (Karimi et al., 2024). In animals, the historical use of FMT is known as ‘transfaunation’, which is primarily used in ruminants to restore microbial populations in the rumen, addressing digestive and metabolic disorders (DePeters and George, 2014). The origins of transfaunation can be traced back to the 17th-century in Italy, where it was first documented as a method for restoring normal rumination (Borody et al., 2004). In recent years, interest in FMT has expanded beyond ruminants to include other livestock and companion animals, both for therapeutic and preventive purposes. For instance, FMT has been successfully used to mitigate porcine circovirus-associated disease in piglets, treat canine parvovirus in dogs, and manage colitis in horses (Mullen et al., 2018; Niederwerder, 2018; Pereira et al., 2018). The effectiveness of FMT has also been demonstrated in wildlife veterinary medicine, aiding animal recovery after antibiotic treatments (Bornbusch et al., 2024).

The growing interest in microbiome therapies is largely driven by the increasing prevalence of chronic diseases linked to microbiome imbalances. Just as the gut microbiome is essential for overall health, the microbiomes of the vagina and uterus are crucial for reproductive well-being and fertility. While extensive research on FMT has provided valuable insights into the potential of microbiome therapies, vaginal microbiota transplantation (VMT) is less established and still in the early stages of research. Studies using rat models of vaginal dysbiosis (or vaginosis) suggest that VMT may be effective for reducing inflammation, promoting *Lactobacillus* proliferation, and mitigating symptoms. In humans, the first case study of VMT was conducted in 2019 with five patients, exploring its use as a therapeutic alternative for women with symptomatic, recurrent, and intractable vaginosis (Lev-Sagie et al., 2019). Standard treatment for vaginosis typically involves antibiotics, either systemic or vaginal. However, severe cases can relapse at rates as high as 50-70% within one year (Bradshaw et al., 2006; Wu et al., 2022). In such instances, maintenance antimicrobial treatment is often recommended, but this can increase the risk of vaginal candidiasis (Shukla and Sobel, 2019). While probiotic treatments with oral or vaginal administration of *Lactobacillus* strains show mixed results in the literature, a proof-of-concept study demonstrated promising, long-lasting symptom improvements after single or repeated VMT in four out of five patients with vaginosis. Recently, a study of a 30-year-old woman with recurrent vaginosis for nine years and a history of three late pregnancy losses confirmed that VMT successfully eradicated the condition, leading to a sustained, long-term shift in the vaginal microbiome toward a more balanced state. Notably, five months post-transplantation, the patient conceived and then delivered a healthy baby at full term (Wrønding et al., 2023). Remarkably, this outcome was achieved without prior antibiotic treatment, underscoring the potential of microbiome transplants for treating vaginosis and other chronic diseases associated with microbiome imbalances in the reproductive tract. This approach is particularly beneficial as it avoids the risks of antibiotic resistance and minimizes disruption to other microbiomes, such as the gut.

The potential benefits of direct uterine microbiome transplant (UMT) over VMT in treating key RTID—such as metritis and endometritis in dairy cows, PBIE in mares, postpartum uterine infections in sows, and pyometra in bitches and queens—should be carefully evaluated. To date, no published studies have explored the application of VMT or UMT in these domestic species. Existing research is limited to preliminary studies in rats, where VMT led to shifts in the uterine microbiota composition, and transplants from women with chronic endometritis induced inflammation-like lesions in the rat endometrial tissue (Wang et al., 2021). These findings raise important questions about the potential risks and benefits of VMT in reproductive health, particularly in species with differing reproductive physiology.

Physiological differences in reproductive cycles across species may significantly impact the effectiveness of microbiome transplants in treating RTID associated with microbiome dysbiosis. In women, the cervix remains tightly closed except during menstruation, ovulation, and childbirth, allowing for microbial transfer between the vaginal and uterine microbiomes at these time points. In contrast, in cows, mares, and sows, the cervix opens only during oestrus or parturition, with the duration of oestrus varying by species. Mares experience a prolonged oestrus lasting 5-7 days, whereas in cows and sows, it is much shorter—typically one to two days. Additionally, species with extended dioestrus phases, such as dogs and cats, undergo oestrous cycles only about twice a year, meaning the cervix opens for microbial colonization from the vagina to the uterus only during these infrequent periods. These variations underscore the importance of species-specific considerations when designing experimental studies on reproductive microbiome transplantation. Understanding these factors will be essential in determining whether UMT provides a more targeted and effective approach than VMT for treating RTID in domestic animals.

### Risks and challenges of ‘natural’ microbiome transplantation

Apart from the physiological differences in reproductive cycles among species, several other challenges must be considered when performing reproductive tract microbiome transplants. One major concern is that, despite being sourced from healthy individuals, microbiome transplants often contain unidentified species that may be pathogenic, potentially exacerbating disease symptoms (Bokoliya et al., 2021). Additionally, they pose a risk of infection or triggering an excessive immune response, particularly in immunocompromised recipients (Carlson, 2020; Merrick et al., 2020). Therefore, prior to the treatment an extensive screening process is essential to assess the safety and risks associated with the microbial composition of donor samples. The procedure involves metagenomic sequencing, culture-based pathogen screening, and functional assays to detect potential harmful species. Furthermore, donor selection criteria must account for factors such as age, reproductive status, and antibiotic exposure history, as these can influence microbiome composition and transplant efficacy (Merrick et al., 2020). Beyond safety concerns, another challenge is the ecological compatibility of the transplanted microbiome. The recipient’s native microbial community, immune system, and reproductive environment may not support the engraftment of donor microbes, leading to poor colonization or transient effects (Smillie et al., 2018). For instance, multiple studies have reported unpredictable patient outcomes following FMT, largely due to the complex and poorly understood ecological interactions between donor and recipient microbiomes (Kazemian et al., 2020). Furthermore, the standardization of microbiome transplant protocols remains a critical issue, particularly in veterinary applications where interspecies variability adds another layer of complexity. Factors such as the optimal route of administration (e.g., intrauterine vs. vaginal infusion), dosage, stage of the oestrus cycle, and frequency of transplantation require further investigation.

Another major concern is the use of antibiotics prior to microbiome transplantation. Although transplants are typically performed one to two days after the final antibiotic dose to mitigate residual effects, leftover antibiotics in recipients can still impact the engraftment of donor bacteria (Singh et al., 2022). Furthermore, in the context of FMT antibiotic cocktails are commonly administered orally to deplete the pathogen load and diminish the existing dysbiosis, thereby creating a more favourable environment for the transplanted healthy microbiota. However, this approach fails to account for its systemic effects on microbial communities in other body sites, including the reproductive tract (Wang et al., 2024). Furthermore, antibiotic use prior to microbiome transplantation raises concerns regarding the horizontal transfer of antibiotic resistance genes (ARGs), which may facilitate the dissemination of resistant pathogens (Tan et al., 2025). These risks underscore the complex interplay between the benefits and drawbacks of antibiotic pretreatment. While VMT without prior antibiotic administration has demonstrated efficacy in treating vaginosis (Wrønding et al., 2023), evidence from a meta-analysis suggests that in patients with ulcerative colitis, antibiotic pretreatment may enhance the efficacy of FMT (Keshteli et al., 2017).

Challenges associated with microbiome transfers have also impacted study designs. Ethical concerns and biosafety considerations have led to restrictions on clinical applications, as seen in the rejection of VMT for human use due to concerns about microbiota superinfection or transmission of infectious agents such as HIV. Consequently, researchers have had to adapt their approaches, often limiting initial investigations to animal models before seeking approval for human trials (Chen et al., 2021) .

To overcome these challenges associated with microbiome transfers, two primary strategies are being pursued. Firstly, rigorous clinical trials, such as NCT04046900 (NIH, 2025a), a randomized trial of VMT to restore a *Lactobacillus* dominant vaginal microbial community in women with recurrent vaginosis, are being conducted to systematically evaluate the safety, efficacy, and long-term consequences of microbiome-based therapies. These studies are crucial in establishing standardized protocols, optimizing donor selection criteria, and identifying risk mitigation strategies to enhance the clinical translation of microbiome transplants. Secondly, researchers are investigating the development of synthetic or engineered microbiomes as a more controlled, standardized, and predictable alternative to traditional microbiome transplantation. The potential benefits and methodologies of synthetic microbiomes will be explored in the following section.

### Synthetic microbiome transplantation: a future solution?

The challenges associated with microbiome transplantation, combined with the inherent complexity and variability of host-associated microbiomes, have driven scientists to explore an alternative approach: the development of synthetic microbial communities designed to replicate the composition and functionality of their natural counterparts (Großkopf and Soyer, 2014). These engineered consortia offer several advantages over donor-derived microbiome transplants (Table 2). Furthermore, not only can they provide more controlled and standardized therapeutic applications, but they also serve as powerful model systems for studying the functional, ecological, and structural dynamics of native microbiota.

**Table 2:** Comparison of key features of natural versus synthetic microbiome transplants.

**Feature**	**Natural microbiome transplant (Donor-Derived)**	**Synthetic microbiome transplant (Bioreactor-Grown)**
**Diversity**	Highly diverse, contains full microbial ecosystem (including unknown/uncharacterized species)	Limited to selected strains, may lack some functional interactions
**Efficacy**	More likely to establish a stable microbiome due to presence of native microbial networks	May require optimization to achieve colonization and stability
**Safety**	Risk of pathogen transfer (e.g., viruses, antibiotic-resistant bacteria)	Free from pathogens if produced under controlled conditions
**Standardization**	Variable between donors, difficult to ensure consistency	Highly controlled and reproducible
**Regulatory Approval**	More challenging due to donor variability and screening requirements	Easier to obtain approval due to known composition
**Production & Scalability**	Requires continuous donor screening and collection	Can be mass-produced under sterile conditions
**Ethical Considerations**	Concerns about donor selection and consent	No ethical concerns related to donor sourcing

Research on synthetic microbiome transplantation in reproductive tract health remains scarce, even in human and mouse models. One study using a *Gardnerella vaginalis*-induced vaginosis mouse model demonstrated that a synthetic microbial consortium, composed of four lactic acid bacteria isolated from the vaginas of healthy women, effectively mitigated vaginal tissue damage, facilitated microbiota restoration, reduced pro-inflammatory cytokine secretion (IL-1β and IL-8), and inhibited NF-κB activation (Li et al., 2023). Neutrophils and macrophages play a central role in responding to microbial dysbiosis by producing cytokines such as IL-1β and IL-8, which mediate immune cell recruitment and inflammatory responses. Suppressing these cytokines reduces excessive inflammation, which is crucial for preventing tissue damage and restoring homeostasis. These findings highlight the potential of synthetic microbial consortia in modulating host immune responses related to RTID. However, the study also reported that natural VMT was more effective than synthetic bacterial community transplantation in suppressing *Gardnerella vaginalis*-induced inflammation, suggesting that naturally derived microbiomes may retain functional advantages in immune regulation (Li et al., 2023).

Producing synthetic microbiomes remains a formidable challenge, primarily because it requires a comprehensive understanding of natural microbial communities—a “healthy template.” This is particularly complex in the context of reproductive tract microbiomes in both women and domestic animals, as discussed in this paper. Our current understanding of microbiome complexity extends beyond taxonomic composition; growing evidence suggests that microbial functionality, such as metabolic activity, is more critical than the taxonomic identity of individual species (Krautkramer et al., 2021). As a result, overall microbial abundance may be less important than the functional roles of key community members. This paradigm shift refocuses microbiome research from species-level diversity to the identification of core functional traits that drive ecosystem stability and host interactions. In this context, the widespread functional redundancy among microbes supports a strategic approach to synthetic microbiome design. Instead of replicating entire natural communities, researchers aim to construct minimal microbial consortia that encompass essential functional roles. This approach has already shown success in various domains, such as enhancing plant–microbe interactions (Gonçalves et al., 2023) and developing rationally designed microbiome therapeutics to prevent and treat chronic immune-mediated colitis in a humanized T cell-mediated mouse model (van der Lelie et al., 2021).

Given the complexity of host-microbiome interactions, there is no straightforward answer as to whether natural or synthetic microbiomes are superior for treating RTID. However, as our understanding of microbiome dynamics, interspecies interactions, and host responses deepens, advancements in synthetic microbiome design may enable the precise engineering of microbial communities with optimized therapeutic potential (Shetty et al., 2022). The ability to strictly control composition, dosing, and application protocols may ultimately enhance the safety and reproducibility of synthetic microbiomes, potentially tipping the balance in favour of their use in future therapeutic applications.

## Conclusion and future perspectives

The management of RTID in domestic animals is at a crucial turning point, where the limitations of traditional antibiotics meet the growing potential of therapies based on microbial ecology. While conventional treatments have focused on eliminating pathogens, this review highlights the need to rethink treatment strategies by considering the role of beneficial microbes and ecological balance. The microbiome of the reproductive tract—a complex mix of host immunity, microbial diversity, and environmental factors—holds great promise for treatments that restore health rather than disrupt it. New approaches, such as transplanting beneficial microbes or creating synthetic microbial consortiums, challenge the old idea of seeing microbes only as “pathogens” or “good bacteria.” Instead, they encourage a more complete view of microbial networks in the body. However, this path forward is complicated. Differences across species, such as the short oestrus cycles in cows and the long periods of reproductive inactivity in dogs, require tailored solutions that consider each species’ unique anatomy, hormones, and immune systems. Additionally, translating research from animal models to larger species or pets raises practical and ethical challenges. Potential risks, such as unintended changes to the microbiome, gene transfer, or immune overreaction, stress the need for careful, species-specific safety measures. Future research must shift from simply cataloguing microbes to understanding how they interact, how they affect the immune system, and how they help maintain a healthy reproductive environment. Collaboration among microbiologists, veterinarians, and bioengineers will be key in developing precise treatments, while regulatory bodies must adapt to the new challenges posed by living biologics. Ultimately, moving from antibiotic-based treatments to microbiome-focused strategies requires a shift in how we define success—not just the absence of harmful pathogens, but the restoration of a healthy, balanced ecosystem. Although this transition is ambitious, it supports broader goals of reducing antimicrobial resistance and promoting sustainable agriculture. By embracing these innovations and understanding the delicate balance of ecosystems, veterinary medicine can lead the way in creating treatments that protect animal health, improve food security, and address the growing global crisis of antimicrobial resistance.

## Data Availability

No research data was used.

## References

[B001] Acland HM, Kenney RM (1983). Lesions of contagious equine metritis in mares. Vet Pathol.

[B002] Bäckhed F, Ley RE, Sonnenburg JL, Peterson DA, Gordon JI (2005). Host-Bacterial Mutualism in the Human Intestine. Science.

[B003] Banchi P, Bertero A, Gionechetti F, Corrò M, Spagnolo E, Donato GG, Pallavicini A, Rota A (2024). The vaginal microbiota of healthy female cats. Theriogenology.

[B004] Banchi P, Colitti B, Del Carro A, Corrò M, Bertero A, Ala U, Del Carro A, van Soom A, Bertolotti L, Rota A (2023). Challenging the hypothesis of in utero microbiota acquisition in healthy canine and feline pregnancies at term: preliminary data. Vet Sci.

[B005] Barba M, Martínez-Boví R, Quereda JJ, Mocé ML, Plaza-Dávila M, Jiménez-Trigos E, Gómez-Martín Á, González-Torres P, Carbonetto B, García-Roselló E (2020). Vaginal microbiota is stable throughout the estrous cycle in arabian mares. Animals.

[B006] Beckwith-Cohen B, Koren O, Blum S, Elad D (2012). Variations in vaginal pH in dairy cattle associated with parity and the periparturient period. Isr J Vet Med.

[B007] Bicalho RC, Santos TMA, Gilbert RO, Caixeta LS, Teixeira LM, Bicalho MLS, Machado VS (2010). Susceptibility of *Escherichia coli* isolated from uteri of postpartum dairy cows to antibiotic and environmental bacteriophages. Part I: isolation and lytic activity estimation of bacteriophages. J Dairy Sci.

[B008] Bicalho MLS, Machado VS, Higgins CH, Lima FS, Bicalho RC (2017). Genetic and functional analysis of the bovine uterine microbiota. Part I: metritis versus healthy cows. J Dairy Sci.

[B009] Bicalho MLS, Santin T, Rodrigues MX, Marques CE, Lima SF, Bicalho RC (2017). Dynamics of the microbiota found in the vaginas of dairy cows during the transition period: associations with uterine diseases and reproductive outcome. J Dairy Sci.

[B010] Blanchard TL, Varner DD, Schumacher J, Love CC, Brinsko SP, Rigby SL (2003). Manual of equine reproduction..

[B011] Bokoliya SC, Dorsett Y, Panier H, Zhou Y (2021). Procedures for fecal microbiota transplantation in murine microbiome studies. Front Cell Infect Microbiol.

[B012] Bornbusch SL, Crosier A, Gentry L, Delaski KM, Maslanka M, Muletz-Wolz CR (2024). Fecal microbiota transplants facilitate post-antibiotic recovery of gut microbiota in cheetahs (*Acinonyx jubatus*). Commun Biol.

[B013] Borody TJ, Warren EF, Leis SM, Surace R, Ashman O, Siarakas S (2004). Bacteriotherapy using fecal flora: toying with human motions. J Clin Gastroenterol.

[B014] Bradshaw CS, Morton AN, Hocking J, Garland SM, Morris MB, Moss LM, Horvath LB, Kuzevska I, Fairley CK (2006). High recurrence rates of bacterial vaginosis over the course of 12 months after oral metronidazole therapy and factors associated with recurrence. J Infect Dis.

[B015] Canisso IF, Segabinazzi LGTM, Fedorka CE (2020). Persistent breeding-induced endometritis in mares: a multifaceted challenge: from clinical aspects to immunopathogenesis and pathobiology. Int J Mol Sci.

[B016] Carlson PE (2020). Regulatory considerations for fecal microbiota transplantation products. Cell Host Microbe.

[B017] Carneiro LC, Cronin JG, Sheldon IM (2016). Mechanisms linking bacterial infections of the bovine endometrium to disease and infertility. Reprod Biol.

[B018] Carvalho AC, Barbosa AV, Arais LR, Ribeiro PF, Carneiro VC, Cerqueira AMF (2016). Resistance patterns, ESBL genes, and genetic relatedness of *Escherichia coli* from dogs and owners. Braz J Microbiol.

[B019] Chen T, Xia C, Hu H, Wang H, Tan B, Tian P, Zhao X, Wang L, Han Y, Deng KY, Wei H, Xin HB (2021). Dysbiosis of the rat vagina is efficiently rescued by vaginal microbiota transplantation or probiotic combination. Int J Antimicrob Agents.

[B020] Chenault JR, McAllister JF, Chester ST, Dame KJ, Kausche FM, Robb EJ (2004). Efficacy of ceftiofur hydrochloride sterile suspension administered parenterally for the treatment of acute postpartum metritis in dairy cows. J Am Vet Med Assoc.

[B021] Claeyé E, Beek J, Meyns T, Maes D (2015). Effect of ketoprofen treatment in the prevention of postpartum dysgalactia syndrome in sows. Vlaams Diergeneeskd Tijdschr.

[B022] Coggan J, Melville P, Oliveira C, Faustino M, Moreno A, Benites N (2008). Microbiological and histopathological aspects of canine pyometra. Braz J Microbiol.

[B023] Çömlekcioğlu U, Jezierska S, Opsomer G, Pascottini OB (2024). Uterine microbial ecology and disease in cattle: A review. Theriogenology.

[B024] Dascanio JJ, Schweizer C, Ley WB (2001). Equine fungal endometritis. Equine Vet Educ.

[B025] De Bosschere H, Ducatelle R, Vermeirsch H, van den Broeck W, Coryn M (2001). Cystic endometrial hyperplasia- pyometra complex in the bitch: should the two entities be disconnected?. Theriogenology.

[B026] Deguillaume L, Geffré A, Desquilbet L, Dizien A, Thoumire S, Vornière C, Constant F, Fournier R, Chastant-Maillard S (2012). Effect of endocervical inflammation on days to conception in dairy cows. J Dairy Sci.

[B027] Del Prete C, Montano C, Cocchia N, de Chiara M, Gasparrini B, Pasolini MP (2024). Use of regenerative medicine in the treatment of endometritis in mares: A systematic review and meta-analysis. Theriogenology.

[B028] Denis-Robichaud J, Dubuc J (2015). Randomized clinical trial of intrauterine cephapirin infusion in dairy cows for the treatment of purulent vaginal discharge and cytological endometritis. J Dairy Sci.

[B029] DePeters EJ, George LW (2014). Rumen transfaunation. Immunol Lett.

[B030] Dini P, Farhoodi M, Hostens M, Van Eetvelde M, Pascottini OB, Fazeli MH, Opsomer G (2015). Effect of uterine lavage on neutrophil counts in postpartum dairy cows. Anim Reprod Sci.

[B031] Dubuc J, Duffield TF, Leslie KE, Walton JS, LeBlanc SJ (2010). Definitions and diagnosis of postpartum endometritis in dairy cows. J Dairy Sci.

[B032] Eckel EF, Ametaj BN (2016). Invited review: role of bacterial endotoxins in the etiopathogenesis of periparturient diseases of transition dairy cows. J Dairy Sci.

[B033] Einarsson S, Brandt Y, Lundeheim N, Madej A (2008). Stress and its influence on reproduction in pigs: a review. Acta Vet Scand.

[B034] Escandón BM, Espinoza JS, Perea FP, Quito F, Ochoa R, López GE, Galarza DA, Garzón JP (2020). Intrauterine therapy with ozone reduces subclinical endometritis and improves reproductive performance in postpartum dairy cows managed in pasture-based systems. Trop Anim Health Prod.

[B035] Eshghi D, Kafi M, Sharifiyazdi H, Azari M, Ahmadi N, Ghasrodashti AR, Sadeghi M (2022). Intrauterine infusion of blood serum of dromedary camel improves the uterine health and fertility in high producing dairy cows with subclinical endometritis. Anim Reprod Sci.

[B036] European Union (2019). Regulation (EU) 2019/4 of the European Parliament and of the Council of 11 December 2018 on the manufacture, placing on the market and use of medicated feed, amending Regulation (EC) No 183/2005 of the European Parliament and of the Council and repealing Council Directive 90/167/EEC, L 4/1. Official Journal of the European Union.

[B037] European Union (2019). Regulation (EU) 2019/6 of the European Parliament and of the Council of 11 December 2018 on veterinary medicinal products and repealing Directive 2001/82/EC, L 4/43. Official Journal of the European Union.

[B038] Fernández‐Hernández P, Valero‐González M, Fuentes‐Romero B, Iglesias‐García M, Ezquerra‐Calvo LJ, Martín‐Cuervo M, Macías-García B (2024). Resolution of two cases of ovarian abscesses in mares subjected to ovum pick up. Equine Vet J.

[B039] Ferris RA, McCue PM, Borlee GI, Loncar KD, Hennet ML, Borlee BR (2016). In vitro efficacy of nonantibiotic treatments on biofilm disruption of gram-negative pathogens and an in vivo model of infectious endometritis utilizing isolates from the equine uterus. J Clin Microbiol.

[B040] Filatova AV, Firsov GM, Loshchinin SO, Akhmadov VT, Fayzulina NS (2021). Bacterial and mycotic factors in the pathogenesis of latent endometritis and salpingitis in cows and a decrease in the sanitary quality of milk. BIO Web Conf.

[B041] Fraga M, Perelmuter K, Delucchi L, Cidade E, Zunino P (2008). Vaginal lactic acid bacteria in the mare: evaluation of the probiotic potential of native *Lactobacillus* spp. and *Enterococcus* spp. strains. Antonie van Leeuwenhoek.

[B042] France M, Alizadeh M, Brown S, Ma B, Ravel J (2022). Towards a deeper understanding of the vaginal microbiota. Nat Microbiol.

[B043] Fransson B, Lagerstedt A-S, Hellmen E, Jonsson P (1997). Bacteriological findings, blood chemistry profile and plasma endotoxin levels in bitches with pyometra or other uterine diseases. Zentralbl Veterinärmed A.

[B044] Friso AM, Segabinazzi LGTM, Cyrino M, Correal SB, Freitas-Dell’Aqua CP, Teoro do Carmo M, Dell’Aqua JA, Miró J, Papa FO, Alvarenga MA (2019). Periovulatory administration of firocoxib did not alter ovulation rates and mitigated post-breeding inflammatory response in mares. Theriogenology.

[B045] Genís S, Bach À, Fàbregas F, Arís A (2016). Potential of lactic acid bacteria at regulating *Escherichia coli* infection and inflammation of bovine endometrium. Theriogenology.

[B046] Genís S, Cerri RLA, Bach À, Silper BF, Baylão M, Denis-Robichaud J, Arís A (2018). Pre-calving intravaginal administration of lactic acid bacteria reduces metritis prevalence and regulates blood neutrophil gene expression after calving in dairy cattle. Front Vet Sci.

[B047] Genís S, Sánchez-Chardi A, Bach À, Fàbregas F, Arís A (2017). A combination of lactic acid bacteria regulates *Escherichia coli* infection and inflammation of the bovine endometrium. J Dairy Sci.

[B048] Gholiof M, Adamson-De Luca E, Wessels JM (2022). The female reproductive tract microbiotas, inflammation, and gynecological conditions. Front Reprod Health.

[B049] Gilbert RO (2011). The effects of endometritis on the establishment of pregnancy in cattle. Reprod Fertil Dev.

[B050] Gilbert RO, Santos NR (2016). Dynamics of postpartum endometrial cytology and bacteriology and their relationship to fertility in dairy cows. Theriogenology.

[B051] Golińska E, Sowińska N, Tomusiak-Plebanek A, Szydło M, Witka N, Lenarczyk J, Strus M (2021). The vaginal microflora changes in various stages of the estrous cycle of healthy female dogs and the ones with genital tract infections. BMC Vet Res.

[B052] Gonçalves OS, Creevey CJ, Santana MF (2023). Designing a synthetic microbial community through genome metabolic modeling to enhance plant-microbe interaction. Environ Microbiol.

[B053] Gronsfeld V, Brutinel F, Egyptien S, Porsmoguer C, Hamaide A, Taminiau B, Daube G, Van de Weerdt ML, Deleuze S, Noel S (2024). Evaluation of the vaginal and urinary microbiota of healthy cycling bitches. BMC Vet Res.

[B054] Großkopf T, Soyer OS (2014). Synthetic microbial communities. Curr Opin Microbiol.

[B055] Hashemi A, Mogheiseh A, Ahmadi N, Bigham-Sadegh A, Rajabi A (2024). Unilateral hyperplasia of the oviduct in a dog with a history of normal reproduction. Comp Clin Pathol.

[B056] Heil BA, van Heule M, Thompson SK, Kearns TA, Oberhaus EL, King G, Daels P, Dini P, Sones JL (2023). Effect of sampling method on detection of the equine uterine microbiome during estrus. Vet Sci.

[B057] Heil BA, van Heule M, Thompson SK, Kearns TA, Beckers KF, Oberhaus EL, King G, Daels P, Dini P, Sones JL (2024). Metagenomic characterization of the equine endometrial microbiome during anestrus. J Equine Vet Sci.

[B058] Heuwieser W, Tenhagen B, Tischer M, Lühr J, Blum H (2000). Effect of three programmes for the treatment of endometritis on the reproductive performance of a dairy herd. Vet Rec.

[B059] Holyoak GR, Premathilake HU, Lyman CC, Sones JL, Gunn A, Wieneke X, DeSilva U (2022). The healthy equine uterus harbors a distinct core microbiome plus a rich and diverse microbiome that varies with geographical location. Sci Rep.

[B060] Hurtgen JP (2006). Pathogenesis and treatment of endometritis in the mare: a review. Theriogenology.

[B061] Jeon SJ, Cunha F, Ma X, Martinez N, Vieira-Neto A, Daetz R, Bicalho RC, Lima S, Santos JE, Jeong KC, Galvão KN (2016). Uterine Microbiota and Immune Parameters Associated with Fever in Dairy Cows with Metritis. PLoS One.

[B062] Jeon SJ, Cunha F, Vieira-Neto A, Bicalho RC, Lima S, Bicalho ML, Galvão KN (2017). Blood as a route of transmission of uterine pathogens from the gut to the uterus in cows. Microbiome.

[B063] Karimi M, Shirsalimi N, Hashempour Z, Salehi Omran H, Sedighi E, Beigi F, Mortezazadeh M (2024). Safety and efficacy of fecal microbiota transplantation (FMT) as a modern adjuvant therapy in various diseases and disorders: a comprehensive literature review. Front Immunol.

[B064] Karstrup CC, Klitgaard K, Jensen TK, Agerholm JS, Pedersen HG (2017). Presence of bacteria in the endometrium and placentomes of pregnant cows. Theriogenology.

[B065] Katila T, Ferreira-Dias G (2022). Evolution of the concepts of endometrosis, post breeding endometritis, and susceptibility of mares. Animals.

[B066] Katila T (1995). Onset and Duration of Uterine Inflammatory Response of Mares after Insemination with Fresh Semen. Biol Reprod.

[B067] Kazemian N, Ramezankhani M, Sehgal A, Khalid FM, Kalkhoran AHZ, Narayan A, Wong GK, Kao D, Pakpour S (2020). The trans-kingdom battle between donor and recipient gut microbiome influences fecal microbiota transplantation outcome. Sci Rep.

[B068] Kemper N (2020). Update on postpartum dysgalactia syndrome in sows. J Anim Sci.

[B069] Keshteli AH, Millan B, Madsen KL (2017). Pretreatment with antibiotics may enhance the efficacy of fecal microbiota transplantation in ulcerative colitis: a meta-analysis. Mucosal Immunol.

[B070] Khan IM, Nassar N, Chang H, Khan S, Cheng M, Wang Z, Xiang X (2024). The microbiota: a key regulator of health, productivity, and reproductive success in mammals. Front Microbiol.

[B071] Knickerbocker JJ, Wiltbank MC, Niswender GD (1988). Mechanisms of luteolysis in domestic livestock. Domest Anim Endocrinol.

[B072] Krautkramer KA, Fan J, Bäckhed F (2021). Gut microbial metabolites as multi-kingdom intermediates. Nat Rev Microbiol.

[B073] Lawler DF, Evans RH, Reimers TJ, Colby ED, Monti KL (1991). Histopathologic features, environmental factors, and serum estrogen, progesterone, and prolactin values associated with ovarian phase and inflammatory uterine disease in cats. Am J Vet Res.

[B074] LeBlanc MM, Causey RC (2009). Clinical and subclinical endometritis in the mare: both threats to fertility. Reprod Domest Anim.

[B075] LeBlanc MM, Neuwirth L, Jones L, Cage C, Mauragis D (1998). Differences in uterine position of reproductively normal mares and those with delayed uterine clearance detected by scintigraphy. Theriogenology.

[B076] LeBlanc MM (2010). Advances in the diagnosis and treatment of chronic infectious and post-mating-induced endometritis in the mare. Reprod Domest Anim.

[B077] LeBlanc MM (2008). Common peripartum problems in the mare. J Equine Vet Sci.

[B078] LeBlanc SJ, Duffield TF, Leslie KE, Bateman KG, Keefe GP, Walton JS, Johnson WH (2002). Defining and diagnosing postpartum clinical endometritis and its impact on reproductive performance in dairy cows. J Dairy Sci.

[B079] LeBlanc SJ, Osawa T, Dubuc J (2011). Reproductive tract defense and disease in postpartum dairy cows. Theriogenology.

[B080] Lee YK, Nomoto K, Salminen S, Gorbach SL (1999). Handbook of probiotics..

[B081] Lefebvre RC, Stock AE (2012). Therapeutic efficiency of antibiotics and prostaglandin F2α in postpartum dairy cows with clinical endometritis: an evidence-based evaluation. Vet Clin North Am Food Anim Pract.

[B082] Lev-Sagie A, Goldman-Wohl D, Cohen Y, Dori-Bachash M, Leshem A, Mor U, Strahilevitz J, Moses AE, Shapiro H, Yagel S, Elinav E (2019). Vaginal microbiome transplantation in women with intractable bacterial vaginosis. Nat Med.

[B083] Li Z, Hu Y, Yang Y, Lu Z, Wang Y (2018). Antimicrobial resistance in livestock: antimicrobial peptides provide a new solution for a growing challenge. Anim Front.

[B084] Li B, Yang N, Shan Y, Wang X, Hao Y, Mao R, Teng D, Fan H, Wang J (2020). Therapeutic potential of a designed CSαβ peptide ID13 in *Staphylococcus aureus*-induced endometritis of mice. Appl Microbiol Biotechnol.

[B085] Li Y, Ma X, Yang J, Wu X, Yan Z, He B (2021). Expression Pattern of cathelicidins in dairy cows during endometritis and role of bovine endometrial epithelial cells in production of cathelicidins. Front Vet Sci.

[B086] Li Y, Zhu W, Jiang Y, Lessing DJ, Chu W (2023). Synthetic bacterial consortia transplantation for the treatment of Gardnerella vaginalis-induced bacterial vaginosis in mice. Microbiome.

[B087] Liang H, Cai R, Li C, Glendon OHM, Chengcheng H, Yan H (2022). High-throughput sequencing of 16S rRNA gene analysis reveals novel taxonomic diversity among vaginal microbiota in healthy and affected sows with endometritis. Res Vet Sci.

[B088] Lietaer L, Bogado Pascottini O, Hernandez-Sanabria E, Kerckhof F-M, Lacoere T, Boon N, Vlaminck L, Opsomer G, Van de Wiele T (2021). Low microbial biomass within the reproductive tract of mid-lactation dairy cows: a study approach. J Dairy Sci.

[B089] Lietaer L, Pascottini OB, Lacoere T, Kerckhof F-M, Martens A, Van de Wiele T, Opsomer G (2023). Studying the pre-implantation uterine microbiota in cattle using transabdominal laparoscopic low-volume lavage: aiming for zero-contamination. J Microbiol Methods.

[B090] Lima FS, Vieira-Neto A, Vasconcellos GSFM, Mingoti RD, Karakaya E, Solé E, Bisinotto RS, Martinez N, Risco CA, Galvão KN, Santos JE (2014). Efficacy of ampicillin trihydrate or ceftiofur hydrochloride for treatment of metritis and subsequent fertility in dairy cows. J Dairy Sci.

[B091] Liu IKM, Troedsson MHT (2008). The diagnosis and treatment of endometritis in the mare: yesterday and today. Theriogenology.

[B092] Loncar KD, Ferris RA, McCue PM, Borlee GI, Hennet ML, Borlee BR (2017). In vitro biofilm disruption and bacterial killing using nonantibiotic compounds against gram-negative equine uterine pathogens. J Equine Vet Sci.

[B093] Lyman CC, Holyoak GR, Meinkoth K, Wieneke X, Chillemi KA, DeSilva U (2019). Canine endometrial and vaginal microbiomes reveal distinct and complex ecosystems. PLoS One.

[B094] Machado VS, Bicalho MLS, Pereira RV, Caixeta LS, Bittar JHJ, Oikonomou G, Gilbert RO, Bicalho RC (2012). The effect of intrauterine administration of mannose or bacteriophage on uterine health and fertility of dairy cows with special focus on *Escherichia coli* and *Arcanobacterium pyogenes*. J Dairy Sci.

[B095] Machado VS, Oikonomou G, Bicalho MLS, Knauer WA, Gilbert R, Bicalho RC (2012). Investigation of postpartum dairy cows’ uterine microbial diversity using metagenomic pyrosequencing of the 16S rRNA gene. Vet Microbiol.

[B096] Machado VS, Silva TH (2020). Adaptive immunity in the postpartum uterus: potential use of vaccines to control metritis. Theriogenology.

[B097] Madureira AML, Burnett TA, Boyd CT, Baylão M, Cerri RLA (2023). Use of intravaginal lactic acid bacteria prepartum as an approach for preventing uterine disease and its association with fertility of lactating dairy cows. J Dairy Sci.

[B098] Malaluang P, Åkerholm T, Nyman G, Lindahl J, Hansson I, Morrell JM (2024). Bacteria in the healthy equine vagina during the estrous cycle. Theriogenology.

[B099] Marchant JN, Rudd AR, Mendl MT, Broom DM, Meredith MJ, Corning S, Simmins PH (2000). Timing and causes of piglet mortality in alternative and conventional farrowing systems. Vet Rec.

[B100] Meira EBS, Rossi RS, Teixeira AG, Kaçar C, Oikonomou G, Gregory L, Bicalho RC (2013). The effect of prepartum intravaginal bacteriophage administration on the incidence of retained placenta and metritis. J Dairy Sci.

[B101] Merrick B, Allen L, Masirah M, Zain N, Forbes B, Shawcross DL, Goldenberg SD (2020). Regulation, risk and safety of faecal microbiota transplant. Infect Prev Pract.

[B102] Metcalf ES (2014). The effect of Platelet-Rich Plasma (PRP) on intraluminal fluid and pregnancy rates in mares susceptible to Persistent Mating-Induced Endometritis (PMIE). J Equine Vet Sci.

[B103] Milani C, Corrò M, Drigo M, Rota A (2012). Antimicrobial resistance in bacteria from breeding dogs housed in kennels with differing neonatal mortality and use of antibiotics. Theriogenology.

[B104] Miranda-CasoLuengo R, Lu J, Williams EJ, Miranda-CasoLuengo AA, Carrington SD, Evans ACO, Meijer WG (2019). Delayed differentiation of vaginal and uterine microbiomes in dairy cows developing postpartum endometritis. PLoS One.

[B105] Moore SG, Ericsson AC, Poock SE, Melendez P, Lucy MC (2017). Hot topic: 16S rRNA gene sequencing reveals the microbiome of the virgin and pregnant bovine uterus. J Dairy Sci.

[B106] Moraes JGN, Gull T, Ericsson AC, Poock SE, Caldeira MO, Lucy MC (2024). Establishment of the uterine microbiome following artificial insemination in virgin heifers. Front Microbiol.

[B107] Moraes JGN, Gull T, Ericsson AC, Poock SE, Caldeira MO, Lucy MC (2024). The microbiome of the pregnant uterus in Holstein dairy heifers and cows assessed by bacterial culture and 16S ribosomal RNA gene sequencing. Front Microbiol.

[B108] Morris HAL, McCue MP, Aurich C (2020). Equine endometritis: a review of challenges and new approaches. Reproduction.

[B109] Mullen KR, Yasuda K, Divers TJ, Weese JS (2018). Equine faecal microbiota transplant: current knowledge, proposed guidelines and future directions. Equine Vet Educ.

[B110] Naddaf M (2024). Antibiotic resistance could cause 40 million deaths by 2025. Nature.

[B111] NIH (2025). Vaginal microbiota transplant (MOTIF).

[B112] NIH (2025). Human microbiome project.

[B113] Nebel-Karp A, Mahoney D, Whitacre M, Bauer B, Lyle S (2025). Infertility caused by oophoritis in a dog resolved by hemiovariectomy. Clin Theriogenology.

[B114] Niederwerder MC (2018). Fecal microbiota transplantation as a tool to treat and reduce susceptibility to disease in animals. Vet Immunol Immunopathol.

[B115] Paccamonti D, Pycock J, Noakes DE, Parkinson TJ, England GCW (2009). Veterinary reproduction and obstetrics..

[B116] Paiano RB, Baruselli PS (2022). The use of herbal treatments as alternatives to control uterine diseases in dairy cows. Trop Anim Health Prod.

[B117] Paiano RB, Morrison EI, LeBlanc Stephen J (2024). Randomized clinical trial of ketoprofen or ceftiofur for treatment of metritis in dairy cows. J Dairy Sci.

[B118] Pascottini OB, LeBlanc SJ (2020). Modulation of immune function in the bovine uterus peripartum. Theriogenology.

[B119] Pascottini OB, Van Schyndel SJ, Spricigo JFW, Rousseau J, Weese JS, LeBlanc SJ (2020). Dynamics of uterine microbiota in postpartum dairy cows with clinical or subclinical endometritis. Sci Rep.

[B120] Pascottini OB, Aurich C, England G, Grahofer A (2023). General and comparative aspects of endometritis in domestic species: a review. Reprod Domest Anim.

[B121] Pascottini OB, LeBlanc SJ, Gnemi G, Leroy JLMR, Opsomer G (2023). Genesis of clinical and subclinical endometritis in dairy cows. Reproduction.

[B122] Pelzer ES, Allan JA, Waterhouse MA, Ross T, Beagley KW, Knox CL (2013). Microorganisms within human follicular fluid: effects on IVF. PLoS One.

[B123] Pereira GQ, Gomes LA, Santos IS, Alfieri AF, Weese JS, Costa MC (2018). Fecal microbiota transplantation in puppies with canine parvovirus infection. J Vet Intern Med.

[B124] Płoneczka-Janeczko K, Magdziarz M, Siemieniuch-Tartanus M (2024). The vaginal microbiome of mares on the post-foaling day under field conditions. Animals.

[B125] Plush KJ, Pluske JR, Lines DS, Ralph CR, Kirkwood RN (2021). Meloxicam and dexamethasone administration as anti-inflammatory compounds to sows prior to farrowing does not improve lactation performance. Animals.

[B126] Pohl A, Bertulat S, Borchardt S, Burfeind O, Heuwieser W (2016). Randomized, controlled clinical trial on the efficacy of nonsteroidal antiinflammatory drugs for the treatment of acute puerperal metritis in dairy cows. J Dairy Sci.

[B127] Poor AP, Moreno LZ, Monteiro MS, Matajira CEC, Dutra MC, Leal DF, Silva APS, Gomes VTM, Barbosa MRF, Sato MIZ, Moreno AM (2022). Vaginal microbiota signatures in healthy and purulent vulvar discharge sows. Sci Rep.

[B128] Quereda JJ, Barba M, Mocé ML, Gomis J, Jiménez-Trigos E, García-Muñoz Á, Gómez-Martín Á, González-Torres P, Carbonetto B, García-Roselló E (2020). Vaginal Microbiota Changes During Estrous Cycle in Dairy Heifers. Front Vet Sci.

[B129] Rashid MH, Pascottini OB, Xie L, Niazi M, Lietaer L, Comlekcioglu U, Opsomer G (2025). Shotgun metagenomic composition, microbial interactions and functional insights into the uterine microbiome of postpartum dairy cows with clinical and subclinical endometritis. Sci Rep.

[B130] Rebordão M, Galvão A, Szóstek A, Amaral A, Mateus L, Skarzynski D, Ferreira-Dias G (2014). Physiopathologic Mechanisms Involved in Mare Endometrosis. Reprod Domest Anim.

[B131] Reghini MFS, Ramires C, Segabinazzi LG, Castro Chaves MMB, Dell’Aqua CP, Bussiere MC, Dell’Aqua JA, Papa FO, Alvarenga MA (2016). Inflammatory response in chronic degenerative endometritis mares treated with platelet-rich plasma. Theriogenology.

[B132] Reilas T, Katila T, Mäkelä O, Huhtinen M, Koskinen E (1997). Intrauterine fluid accumulation in oestrous mares. Acta Vet Scand.

[B133] Rodrigues G, Souza-Santos L, Franco OL (2022). Antimicrobial Peptides Controlling Resistant Bacteria in Animal Production. Front Microbiol.

[B134] Rojer H, Aurich C (2010). Treatment of persistent mating‐induced endometritis in mares with the non‐steroid anti‐inflammatory drug vedaprofen. Reprod Domest Anim.

[B135] Rota A, Corrò M, Patuzzi I, Milani C, Masia S, Mastrorilli E, Petrin S, Longo A, Del Carro A, Losasso C (2020). Effect of sterilization on the canine vaginal microbiota: a pilot study. BMC Vet Res.

[B136] Sadeghi M, Azari M, Kafi M, Nourani H, Ghaemi M, Najafi M, Eshghi D (2022). Bovine salpingitis: Histopathology, bacteriology, cytology and transcriptomic approaches and its impact on the oocyte competence. Anim Reprod Sci.

[B137] Saltiel A, Páramo R, Murcia C, Tolosa J (1986). Pathologie findings in the oviducts of mares. Am J Vet Res.

[B138] Santos TMA, Gilbert RO, Caixeta LS, Machado VS, Teixeira LM, Bicalho RC (2010). Susceptibility of *Escherichia coli* isolated from uteri of postpartum dairy cows to antibiotic and environmental bacteriophages. Part II: in vitro antimicrobial activity evaluation of a bacteriophage cocktail and several antibiotics. J Dairy Sci.

[B139] Schnobrich M (2019). A review of the equine oviduct: pathology, evaluation, and current treatments. Clin Theriogenology.

[B140] Sheldon IM, Lewis GS, LeBlanc S, Gilbert RO (2006). Defining postpartum uterine disease in cattle. Theriogenology.

[B141] Sheldon IM, Noakes DE, Parkinson TJ, England GCW (2019). Veterinary reproduction and obstetrics..

[B142] Shetty SA, Kostopoulos I, Geerlings SY, Smidt H, de Vos WM, Belzer C (2022). Dynamic metabolic interactions and trophic roles of human gut microbes identified using a minimal microbiome exhibiting ecological properties. ISME J.

[B143] Shukla A, Sobel JD (2019). Vulvovaginitis caused by candida species following antibiotic exposure. Curr Infect Dis Rep.

[B144] Silva JA, Castañares M, Mouguelar H, Valenciano JA, Pellegrino MS (2024). Isolation of lactic acid bacteria from the reproductive tract of mares as potentially beneficial strains to prevent equine endometritis. Vet Res Commun.

[B145] Singh P, Alm EJ, Kelley JM, Cheng V, Smith M, Kassam Z, Nee J, Iturrino J, Lembo A (2022). Effect of antibiotic pretreatment on bacterial engraftment after Fecal Microbiota Transplant (FMT) in IBS-D. Gut Microbes.

[B146] Smillie CS, Sauk J, Gevers D, Friedman J, Sung J, Youngster I, Hohmann EL, Staley C, Khoruts A, Sadowsky MJ, Allegretti JR, Smith MB, Xavier RJ, Alm EJ (2018). Strain tracking reveals the determinants of bacterial engraftment in the human gut following fecal microbiota transplantation. Cell Host Microbe.

[B147] Sonnenburg JL, Angenent LT, Gordon JI (2004). Getting a grip on things: how do communities of bacterial symbionts become established in our intestine?. Nat Immunol.

[B148] Stojanović N, Plećaš D, Plešinac S (2012). Normal vaginal flora, disorders and application of probiotics in pregnancy. Arch Gynecol Obstet.

[B149] Subramanyam PNB, Yeddula S, Baindara P, Mandal SM (2024). Evolution of antimicrobial peptides..

[B150] Swartz JD, Lachman M, Westveer K, O’Neill T, Geary T, Kott RW, Berardinelli JG, Hatfield PG, Thomson JM, Roberts A, Yeoman CJ (2014). Characterization of the vaginal microbiota of ewes and cows reveals a unique microbiota with low levels of lactobacilli and near-neutral pH. Front Vet Sci.

[B151] Tan R, Jin M, Li J, Yang D (2025). The dissemination, health risks, and mitigation approaches of antibiotic resistance genes in the gut microbiome. J Hazard Mater Adv.

[B152] Timoney PJ, Powell DG (1988). Contagious equine metritis: epidemiology and control. J Equine Vet Sci.

[B153] Troedsson MHT (2008). Problems After Breeding. J Equine Vet Sci.

[B154] Troedsson MHT (1997). Therapeutic considerations for mating-induced endometritis. Pferdeheilkunde.

[B155] van der Lelie D, Oka A, Taghavi S, Umeno J, Fan T-J, Merrell KE, Watson SD, Ouellette L, Liu B, Awoniyi M, Lai Y, Chi L, Lu K, Henry CS, Sartor RB (2021). Rationally designed bacterial consortia to treat chronic immune-mediated colitis and restore intestinal homeostasis. Nat Commun.

[B156] van Heule M, Monteiro HF, Bazzazan A, Scoggin K, Rolston M, El-Sheikh Ali H, Weimer BC, Ball B, Daels P, Dini P (2023). Characterization of the equine placental microbial population in healthy pregnancies. Theriogenology.

[B157] Verstegen J, Dhaliwal G, Verstegen-Onclin K (2008). Mucometra, cystic endometrial hyperplasia, and pyometra in the bitch: advances in treatment and assessment of future reproductive success. Theriogenology.

[B158] Virendra A, Gulavane SU, Ahmed ZA, Reddy R, Chaudhari RJ, Gaikwad SM, Shelar RR, Ingole SD, Thorat VD, Khanam A, Khan FA (2024). Metagenomic analysis unravels novel taxonomic differences in the uterine microbiome between healthy mares and mares with endometritis. Vet Med Sci.

[B159] Wagener K, Gabler C, Drillich M (2017). A review of the ongoing discussion about definition, diagnosis and pathomechanism of subclinical endometritis in dairy cows. Theriogenology.

[B160] Walker C, LeBlanc MM (2008). The chronically infertile mare..

[B161] Walker WA, Floch MH, Ringel Y, Walker WA (2017). The microbiota in gastrointestinal pathophysiology: implications for human health, prebiotics, probiotics, and dysbiosis..

[B162] Wang J, Li Z, Ma X, Du L, Jia Z, Cui X, Yu L, Yang J, Xiao L, Zhang B, Fan H, Zhao F (2021). Translocation of vaginal microbiota is involved in impairment and protection of uterine health. Nat Commun.

[B163] Wang ML, Liu MC, Xu J, An LG, Wang JF, Zhu YH (2018). Uterine microbiota of dairy cows with clinical and subclinical endometritis. Front Microbiol.

[B164] Wang Y, Guo H, Bai Y, Li T, Xu R, Sun T, Lu J, Song Q (2020). Isolation and characteristics of multi-drug resistant *Streptococcus porcinus* from the vaginal secretions of sow with endometritis. BMC Vet Res.

[B165] Wang Y, Zhang Z, Chen Q, Chen T (2024). Simultaneous application of oral and intravaginal probiotics for Helicobacter pylori and its antibiotic-therapy-induced vaginal dysbacteriosis. NPJ Biofilms Microbiomes.

[B166] Wang Z, He Y, Zheng Y (2019). Probiotics for the Treatment of Bacterial Vaginosis: A Meta-Analysis. Int J Environ Res Public Health.

[B167] WHO (2024). WHO updates list of drug-resistant bacteria most threatening to human health.

[B168] Wieërs G, Belkhir L, Enaud R, Leclercq S, Philippart de Foy J-M, Dequenne I, de Timary P, Cani PD (2020). How probiotics affect the microbiota. Front Cell Infect Microbiol.

[B169] Williams EJ, Fischer DP, Pfeiffer DU, England GCW, Noakes DE, Dobson H, Sheldon IM (2005). Clinical evaluation of postpartum vaginal mucus reflects uterine bacterial infection and the immune response in cattle. Theriogenology.

[B170] Wrønding T, Vomstein K, Bosma EF, Mortensen B, Westh H, Heintz JE, Mollerup S, Petersen AM, Ensign LM, DeLong K, van Hylckama Vlieg JET, Thomsen AB, Nielsen HS (2023). Antibiotic-free vaginal microbiota transplant with donor engraftment, dysbiosis resolution and live birth after recurrent pregnancy loss: a proof of concept case study. EClinicalMedicine.

[B171] Wu S, Hugerth LW, Schuppe-Koistinen I, Du J (2022). The right bug in the right place: opportunities for bacterial vaginosis treatment. NPJ Biofilms Microbiomes.

[B172] Xu S, Dong Y, Shi J, Li Z, Che L, Lin Y, Li J, Feng B, Fang Z, Yong Z, Wang J, Wu D (2021). Responses of vaginal microbiota to dietary supplementation with lysozyme and its relationship with rectal microbiota and sow performance from late gestation to early lactation. animals.

[B173] Ye G, Huang J, Li G, Zhang J, Sun Y, Zeng D, Bao W, Zhong J, Huang Q (2021). Clinical efficacy of intravaginal recombinant lysostaphin administration on endometritis in sows. Vet Med Sci.

[B174] Ylhäinen A, Mölsa S, Thomson K, Laitinen-Vapaavuori O, Rantala M, Grönthal T (2025). Bacteria associated with canine pyometra and concurrent bacteriuria: a prospective study. Vet Microbiol.

[B175] Zduńczyk S, Janowski T (2020). Bacteriophages and associated endolysins in therapy and prevention of mastitis and metritis in cows: current knowledge. Anim Reprod Sci.

[B176] Zhang J, Liu M, Ke S, Huang X, Fang S, He M, Fu H, Chen C, Huang L (2021). Gut and Vagina Microbiota Associated With Estrus Return of Weaning Sows and Its Correlation With the Changes in Serum Metabolites. Front Microbiol.

[B177] Zheng H, Du C, Zhang Y, Yu C, Huang R, Tang X, Xie GH (2023). A study on the correlation between intrauterine microbiota and uterine pyogenesis in dogs. Theriogenology.

